# Specialized acyl carrier protein used by serine palmitoyltransferase to synthesize sphingolipids in *Rhodobacteria*

**DOI:** 10.3389/fmicb.2022.961041

**Published:** 2022-08-04

**Authors:** Jonathan Padilla-Gómez, Roberto Jhonatan Olea-Ozuna, Sandra Contreras-Martínez, Orlando Morales-Tarré, Daniela A. García-Soriano, Diana X. Sahonero-Canavesi, Sebastian Poggio, Sergio Encarnación-Guevara, Isabel M. López-Lara, Otto Geiger

**Affiliations:** ^1^Centro de Ciencias Genómicas, Universidad Nacional Autónoma de México, Cuernavaca, Mexico; ^2^Instituto de Investigaciones Biomédicas, Universidad Nacional Autónoma de México, Ciudad de México, Mexico

**Keywords:** ACP, acyl-ACP synthetase, fatty acid metabolism, serine palmitoyltransferase, 3-oxo-sphinganine, sphingolipid, *Caulobacter crescentus*, *Escherichia coli* BL21(DE3)

## Abstract

Serine palmitoyltransferase (SPT) catalyzes the first and committed step in sphingolipid biosynthesis condensating L-serine and acyl-CoA to form 3-oxo-sphinganine. Whenever the structural gene for SPT is present in genomes of *Rhodobacteria* (α-, β-, and γ-*Proteobacteria*), it co-occurs with genes coding for a putative acyl carrier protein (ACP) and a putative acyl-CoA synthetase (ACS). In the α-proteobacterium *Caulobacter crescentus*, CC_1162 encodes an SPT, whereas CC_1163 and CC_1165 encode the putative ACP and ACS, respectively, and all three genes are known to be required for the formation of the sphingolipid intermediate 3-oxo-sphinganine. Here we show that the putative ACP possesses a 4'-phosphopantetheine prosthetic group, is selectively acylated by the putative ACS and therefore is a specialized ACP (AcpR) required for sphingolipid biosynthesis in *Rhodobacteria*. The putative ACS is unable to acylate coenzyme A or housekeeping ACPs, but acylates specifically AcpR. Therefore, it is a specialized acyl-ACP synthetase (AasR). SPTs from *C. crescentus*, *Escherichia coli* B, or *Sphingomonas wittichii* use preferentially acyl-AcpR as thioester substrate for 3-oxo-sphinganine synthesis. Whereas acyl-AcpR from *C. crescentus* is a good substrate for SPTs from distinct *Rhodobacteria*, acylation of a specific AcpR is achieved by the cognate AasR from the same bacterium. *Rhodobacteria* might use this more complex way of 3-oxo-sphinganine formation in order to direct free fatty acids toward sphingolipid biosynthesis.

## Introduction

Sphingolipids are essential structural components of eukaryotic membranes, where they play crucial roles in cell signaling and organization of lipid rafts (Nelson and Cox, 2017). They have been implicated in a wide variety of different cellular processes such as cell differentiation, pathogenesis and apoptosis (Hannun and Obeid, 2018). Although sphingolipids are not commonly found in bacteria, they have been identified in *Sphingomonas* (Kawahara et al., 1991; Kawahara et al., 2002) and *Bacteroides* (Kato et al., 1995) species.

The first committed step of the *de novo* biosynthesis of sphingolipids is catalyzed by serine palmitoyltransferase (SPT) and consists in a decarboxylative, Claisen-like condensation of the amino acid L-serine with fatty acyl-coenzyme A (fatty acyl-CoA) in order to obtain 3-oxo-sphinganine, the first intermediate in sphingolipid biosynthesis of eukaryotes (Kerbarh et al., 2006) and bacteria (Brown et al., 2019; Olea-Ozuna et al., 2021). SPT is a member of the α-oxoamine synthase family, which includes enzymes involved in heme and biotin synthesis and requires pyridoxal phosphate. Phylogenetic analysis of this family suggested that the ability to synthesize sphingolipids might be more widespread than previously thought in members of two major phyla of *Bacteria*, the *Bacteroidetes* and the *Proteobacteria* (Geiger et al., 2019). At least two distinct subgroups of bacterial SPTs exist (Geiger et al., 2019). One subgroup includes SPTs of the *Bacteroidetes* (*Bacteroides, Sphingobacterium*, and *Porphyromonas*) and the δ-*Proteobacteria* (*Bacteriovorax stolpii*, *Myxococcus xanthus*, *Stigmatella aurantiaca*, and *Sorangium cellulosum*), whereas the other subgroup includes members of the *Rhodobacteria*, a subphylum of Gram-negative bacteria, that includes α-, β-, and γ-*Proteobacteria*. For example, this latter subgroup of SPTs occurs in the α-*Proteobacteria Caulobacter crescentus*, *Gluconobacter oxydans*, *Sphingomonas wittichii*, *Sphingomonas paucimobilis*, and *Zymomonas mobilis*, in the β-*Proteobacterium Nitrosomonas eutropha*, and in the γ-*Proteobacterium Escherichia coli* B [i.e., in the enterotoxigenic *Escherichia coli* (ETEC) B7A and in the protein expression host *E. coli* BL21(DE3) (Jeong et al., 2015)]. SPTs of the *Rhodobacteria* clearly group together in phylogenetic trees (Geiger et al., 2010, 2019), and their structural genes are usually preceded by a gene predicted to code for an acyl carrier protein (ACP) (Geiger et al., 2010; Raman et al., 2010). *Rhodobacteria* genomes also harbor a well-conserved structural gene for a predicted acyl-CoA synthetase (ACS), frequently in close proximity to the *spt* and the potential *acp* gene.

Acyl carrier proteins (ACPs) are small proteins (<10 kDa) with acidic isoelectric points (pI), carrying a 4'-phosphopantetheine (4'-PPT) prosthetic group to which acyl chains are linked as thioesters during fatty acid or polyketide biosynthesis and transfer. All bacteria possess a constitutively expressed ACP (AcpP), which is needed for essential housekeeping functions and acts as acyl group carrier and donor during fatty acid and membrane lipid biosynthesis (Byers and Gong, 2007). Structural genes for AcpPs (*acpP*) are usually encountered in the genomic neighborhood of other fatty acid biosynthesis (*fab*) genes, i.e., *fabH*, *fabD*, *fabG*, and *fabF* in *E. coli* (Zhang and Cronan, 1996) or *fabD*, *fabG*, and *fabF* in *C. crescentus* (Nierman et al., 2001). Besides their roles in fatty acid, glycerophospholipid, and lipid A biosynthesis, housekeeping AcpPs participate in the synthesis of *N*-acylhomoserine lactone signals for quorum sensing, pore-forming hemolysins, and membrane-derived oligosaccharides (Byers and Gong, 2007). However, some bacteria with complex metabolisms have additional specialized ACPs for the synthesis of secondary metabolites such as polyketides or non-ribosomal peptides (Geiger and López-Lara, 2002; Lai et al., 2006), factors that provoke formation of nitrogen-fixing root nodules on legume host plants by NodF (Ritsema et al., 1998), certain lipid A species by AcpXL (Brozek et al., 1996), and capsular polysaccharides by RkpF (Epple et al., 1998). Although the protein part of ACPs is encoded by their respective structural genes, a post-translational modification is required to convert apo-ACPs into functional holo-ACPs. This reaction is catalyzed by holo-ACP synthase (AcpS) (Lambalot et al., 1996; Flugel et al., 2000) that transfers the 4'-PPT residue from coenzyme A (CoA) to a conserved serine of an ACP.

Fatty acyl-CoA synthetase belongs to the superfamily of adenylate-forming enzymes and plays a central role in intermediary metabolism by catalyzing the formation of fatty acyl-CoA. In *E. coli* this enzyme is encoded by the *fadD* gene and is required for the coupled import and activation of exogenous or endogenous long-chain fatty acids. Fatty acyl-CoAs can then be catabolized by β-oxidation or be reutilized for membrane lipid formation (Sahonero-Canavesi et al., 2019).

Another member of the adenylate-forming enzyme family is acyl-ACP synthetase (Aas). Aas acts on ACPs instead of CoA esterifying fatty acids to the 4'-PPT prosthetic group of holo-ACP coupled to the hydrolysis of ATP. Although Aas was first discovered in *E. coli* (Ray and Cronan, 1976), its use for generating acylated versions of ACPs is limited due to a relatively narrow substrate specificity. Instead, Aas from *Vibrio harveyi* has been developed as an acylating tool, because it has a relatively broad substrate specificity for the type of fatty acids or the type of ACPs (Beld et al., 2014) employed.

**FIGURE 1:**
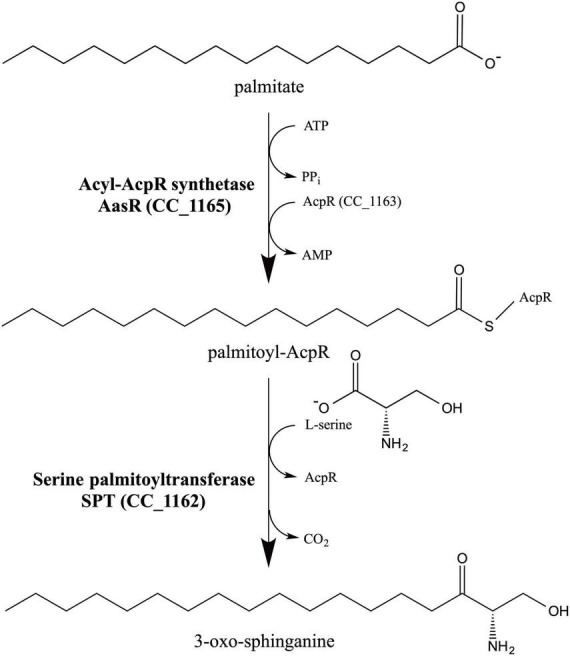
Model for 3-oxo-sphinganine biosynthesis in *C. crescentus*. CC_1165 from *C. crescentus* is a specialized acyl-ACP synthetase (AasR), acylating selectively the specialized acyl carrier protein (AcpR) CC_1163. Subsequently, serine palmitoyltransferase (SPT) CC_1162 from *C. crescentus* uses the specialized acyl-AcpR (CC_1163) as preferred thioester substrate to condense it with L-serine liberating CO_2_ and forming 3-oxo-sphinganine, the first intermediate in sphingolipid biosynthesis. The acyl group represented here is palmitate. We propose naming the specialized ACP as AcpR and specialized acyl-ACP synthetase as AasR because they are both required for ceramide and 3-oxo-sphinganine biosynthesis in *C. crescentus* (Olea-Ozuna et al., 2021) and probably other *Rhodobacteria*.

Studies on sphingolipid biosynthesis in *C. crescentus* revealed that besides *spt* (Stankeviciute et al., 2019), at least four other genes are required for dihydroceramide formation and they were predicted to code for an ACP (CC_1163), a ACS (CC_1165), an epimerase/dehydrogenase (CC_1164), and an acyl-CoA transferase (CC_1154) (Olea-Ozuna et al., 2021). All five of these structural genes are required for fitness (Christen et al., 2011), survival and detergent resistance of *C. crescentus* (Olea-Ozuna et al., 2021). Notably, for the formation of the biosynthetic intermediate 3-oxo-sphinganine in *E. coli*, the combined expression of SPT, the putative ACP, and the putative ACS was required (Olea-Ozuna et al., 2021) and here we propose a biochemical model for 3-oxo-sphinganine synthesis in *C. crescentus* and other *Rhodobacteria* (Figure 1).

Using a combination of labeling experiments and mass spectrometry analyses, we now show that the putative ACP contains a 4'-PPT prosthetic group, can be acylated, and is therefore an ACP. The putative ACS acylates the specialized ACP, but not CoA or the *C. crescentus* housekeeping AcpP, and so constitutes a specialized and selective acyl-ACP synthetase. Furthermore, the acylated specialized ACP is the preferred thioester substrate for SPTs of the *Rhodobacteria* group.

## Results and discussion

### Acyl carrier proteins from *Caulobacter crescentus* and from *Escherichia coli* BL21(DE3) required for sphingolipid biosynthesis contain a 4'-phosphopantetheine prosthetic group

**FIGURE 2:**
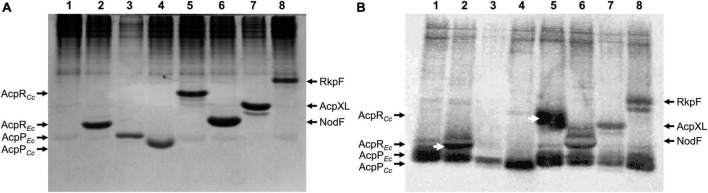
*In vivo* labeling of ACPs with β-[^3^H]alanine. Proteins of cell-free extracts from *E. coli* OG7001 × pLysS strains overproducing different ACPs from pET9a-derived plasmids (Table 1) were separated in a 20% native PAGE. **(A)** Proteins stained with Coomassie blue and **(B)** autoradiogram visualized after labeling with β-[^3^H]alanine. In **(A,B)**, extracts of *E. coli* carrying the pET9a vector (lane 1), AcpR*_*Ec*_* (ECD_02853)-expressing pJPG12 (lane 2), AcpP*_*Ec*_* (ECD_01090)-expressing pTB5079 (lane 3), AcpP*_*Cc*_* (CC_1677)-expressing pPEG01 (lane 4), AcpR*_*Cc*_* (CC_1163)-expressing pDG01 (lane 5), NodF-expressing pMP2301 (lane 6), AcpXL-expressing pAL07 (lane 7) or RkpF-expressing pTB1003 (lane 8) were analyzed. White arrows **(B)** highlight radiolabeled specialized ACPs AcpR*_*Ec*_* (ECD_02853) and AcpR*_*Cc*_* (CC_1163) from *E. coli* and *C. crescentus*, respectively.

Besides the housekeeping AcpP proteins (CC_1677 in *C. crescentus* and ECD_01090 in *E. coli* BL21(DE3)), both bacteria possess a structural gene for putative ACPs (CC_1163 and ECD_02853, respectively), located just upstream of their respective *spt* genes. These four structural *acp* genes were cloned and overexpressed in *E. coli*. Separation of cell-free protein extracts by 20% native polyacrylamide gel electrophoresis (PAGE) and subsequent staining with Coomassie blue (Figure 2A) indicates that ACPs and putative ACPs were readily expressed from all cloned genes (lanes 2–8) and showed characteristic migration behavior. In parallel, *in vivo* labeling experiments with radioactive β-alanine, the biosynthetic precursor of 4'-PPT, were performed with the same strains and after separating cell-free extracts by PAGE, radioactive bands were visualized (Figure 2B). For 4 known ACPs (ECD_01090 = AcpP*_*Ec*_*, lane 3; NodF, lane 6; AcpXL, lane 7; RkpF, lane 8), radiolabeled bands with similar relative mobility as the overexpressed proteins were detected. Also, the putative ACPs (ECD_02853 = AcpR*_*Ec*_*, lane 2; CC_1677 = AcpP*_*Cc*_*, lane 4; CC_1163 = AcpR*_*Cc*_*, lane 5) displayed radiolabeled bands that had similar relative mobility as the overexpressed protein bands (Figure 2), suggesting that in addition to the housekeeping AcpP*_*Cc*_* from *C. crescentus*, the specialized predicted ACPs AcpR*_*Ec*_* from *E. coli* and AcpR*_*Cc*_* from *C. crescentus* also contained β-alanine, the building block of the 4'-PPT prosthetic group characteristic for ACPs. The autoradiogram also reveals that the housekeeping AcpP*_*Ec*_* from *E. coli*, which is present in all samples, including the *E. coli* strain harboring the empty vector pET9a, migrates close to the front and incorporated β-alanine (Figure 2B). In an attempt to obtain a better separation of the distinct ACPs, the same samples analyzed for Figure 1 were also subjected to a SDS-Tricine PAGE (Supplementary Figure 1). Although the housekeeping AcpP*_*Ec*_* from *E. coli* is known to migrate much slower than expected from its molecular weight in SDS-containing gel systems (Rock and Cronan, 1979) and although it is well separated from other ACPs in an SDS-Tricine PAGE (Supplementary Figure 1), all other ACPs migrated similarly, to each other and according to molecular weights of about 10 kDa (Supplementary Figure 1).

### Specialized acyl carrier protein for sphingolipid synthesis from *Caulobacter crescentus* is selectively acylated by specialized acyl-ACP synthetase

To obtain acylated versions of ACPs from *C. crescentus* and *E. coli*, the four distinct ACPs AcpP*_*Cc*_* (CC_1677), AcpR*_*Cc*_* (CC_1163), AcpP*_*Ec*_* (ECD_01090), and AcpR*_*Ec*_* (ECD_02853) were expressed in *E. coli* BL21(DE3) × pLysS and purified (Supplementary Figure 2). Purified ACP preparations contained mixtures of apo- and holo-forms and treatment with the tool of a holo-ACP synthase, as previously described (Ramos-Vega et al., 2009), converted these mixtures into the holo-forms of the respective ACPs (Supplementary Figure 3). Acyl-ACP synthetase (Aas) from *Vibrio harveyi* (Aas*_*Vh*_*) is known for its broad substrate tolerance for many fatty acids and distinct ACPs (Beld et al., 2014). The distinct holo-ACPs were incubated with ATP, fatty acids of various chain lengths and homogenous Aas*_*Vh*_* (Supplementary Figure 4A) and at the end of the assay, potentially acylated versions of the ACPs were separated in a conformationally sensitive PAGE system and visualized by Coomassie blue staining (Supplementary Figure 5). The housekeeping AcpP*_*Ec*_* from *E. coli* (ECD_01090) maintains a more compact structure when acylated with fatty acids and so migrates faster when analyzed by conformationally sensitive PAGE (Beld et al., 2014). When unacylated, AcpP*_*Ec*_* has a less compact structure under the electrophoretic conditions employed and migrates slowly. Acylation with fatty acids of increasing chain lengths causes more compact structures of the acylated AcpP and more rapid migration (Supplementary Figure 5A). Similarly, acylated forms of the housekeeping AcpP from *C. crescentus* (CC_1677) migrate more rapidly in conformationally sensitive PAGE suggesting a more compact form (Supplementary Figure 5B). In the case of specialized AcpR*_*Cc*_*, potentially acylated forms of AcpR*_*Cc*_* might migrate slightly faster than the apo-form and slightly slower than the holo-form (Supplementary Figure 5C). However, as no major change in migration was observed, there is no evidence that AcpR*_*Cc*_* can be acylated by Aas*_*Vh*_*. The potentially different forms of specialized AcpR*_*Ec*_* all migrate in a similar way (Supplementary Figure 5D) and therefore this type of analysis does not permit us to evaluate whether acylation of AcpR*_*Cc*_* or AcpR*_*Ec*_* occurred when Aas*_*Vh*_* was employed as acylating enzyme.

**FIGURE 3:**
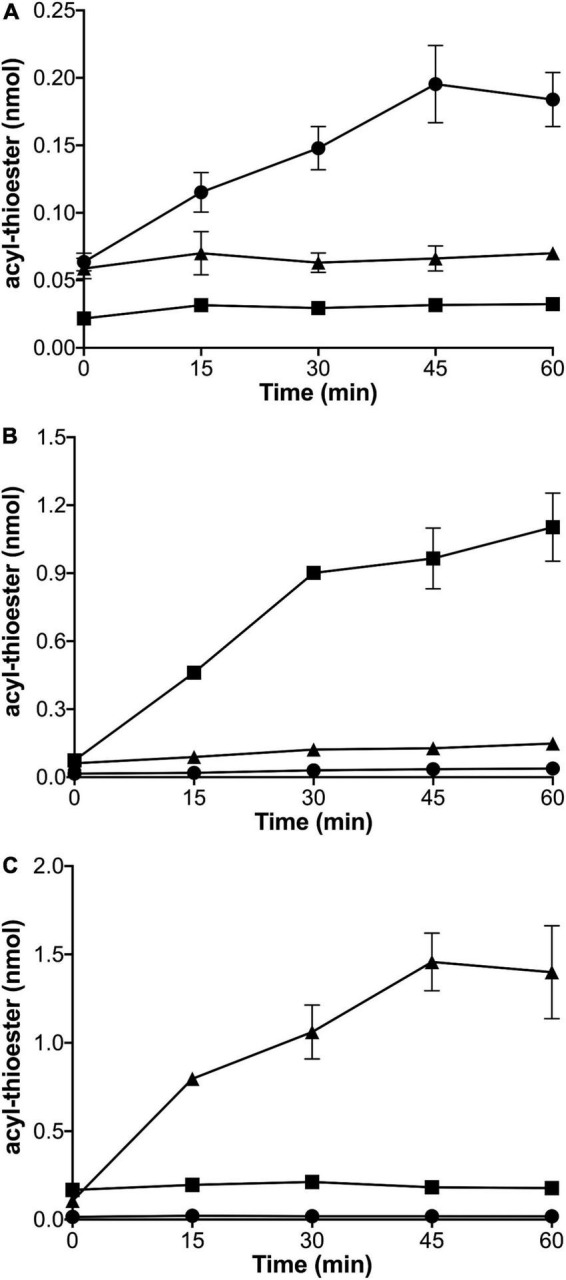
Enzymatic activities of thiol-acylating enzymes AasR*_*Cc*_* from *C. crescentus*, acyl-ACP synthetase Aas*_*Vh*_*, and acyl-CoA synthetase FadD*_*Sm*_*. [^3^H]palmitate incorporated into the acyl-thioester products palmitoyl-AcpR*_*Cc*_* (CC_1163) (•), palmitoyl-AcpP*_*Cc*_* (CC_1677) (■), or palmitoyl-CoA (▲) by AasR*_*Cc*_* (CC_1165) from *C. crescentus* **(A)**, Aas*_*Vh*_* from *V. harveyi* **(B)**, or FadD*_*Sm*_* from *S. meliloti* **(C)**. Reactions were performed at 37°C in a water bath for 0, 15, 30, 45 or 60 min, as described in section “Materials and methods.” In the assay, 1 mM Triton X-100, 40 μM thiol substrate (ACPs or CoA) and different concentrations of each enzyme (30 nM for AasR*_*Cc*_*, 100 nM for Aas*_*Vh*_* and 300 nM for FadD*_*Sm*_*) were used. Standard deviations of three replicates are shown.

To explore which thiol substrates could be acylated by which acylating enzymes, homogeneous preparations of Aas from *V. harveyi* (Aas*_*Vh*_*) (Supplementary Figure 4A), predicted ACS AasR*_*Cc*_* (CC_1165) from *C. crescentus* (Supplementary Figure 4B), and acyl-CoA synthetase from *Sinorhizobium meliloti* (FadD*_*Sm*_*) (Supplementary Figure 4C), were obtained. Each of the homogeneous enzymes was incubated with different potential thiol substrates (CoA, housekeeping AcpP*_*Cc*_*, or specialized AcpR*_*Cc*_*), ATP, and [^3^H]palmitate and at different time points, aliquots were analyzed for radioactivity linked to protein, in the cases of AcpP*_*Cc*_* and AcpR*_*Cc*_*, or to CoA. From the time course for acyl-thioester formation, it is evident that putative ACS AasR*_*Cc*_* from *C. crescentus* caused an increase in the amount of acyl-AcpR*_*Cc*_* whereas no acyl-thioesters were formed when AcpP*_*Cc*_* or CoA were used as substrates (Figure 3A). When different potential thiol substrates were incubated with ATP, [^3^H]palmitate and Aas*_*Vh*_*, an increase in the amount of acyl-AcpP*_*Cc*_* was detected, whereas only very minor increases might have occurred when AcpR*_*Cc*_* or CoA were used as substrates (Figure 3B). Using FadD*_*Sm*_* in the acylation assay resulted in an increase in the amount of acyl-CoA whereas no acyl-thioesters were formed when AcpP*_*Cc*_* or AcpR*_*Cc*_* were used as substrates (Figure 3C). These results clearly show that acyl-CoA synthetase FadD is able to form acyl-CoA but cannot acylate the housekeeping AcpP or specialized ACP AcpR from *C. crescentus*, that the acyl-ACP synthetase Aas from *V. harveyi* is able to acylate the housekeeping AcpP from *C. crescentus*, and that the putative ACS is a specialized acyl-ACP synthetase (AasR) that selectively acylates the specialized AcpR*_*Cc*_* but not CoA or the housekeeping AcpP*_*Cc*_*.

**FIGURE 4:**
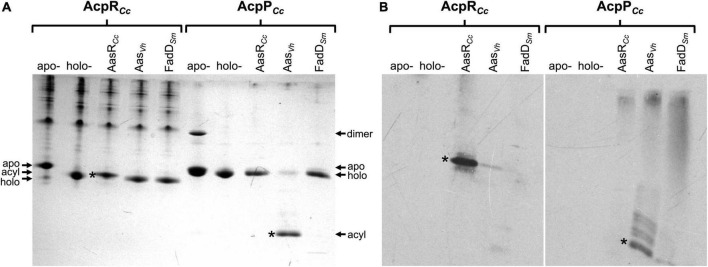
Acylation of specialized AcpR*_*Cc*_* and housekeeping AcpP*_*Cc*_* from *C. crescentus* with [^3^H]palmitate. Palmitate incorporated into palmitoyl-AcpR*_*Cc*_* (CC_1163) or palmitoyl-AcpP*_*Cc*_* (CC_1677) products after incubation with AasR*_*Cc*_* (CC_1165) from *C. crescentus*, Aas from *V. harveyi* (Aas*_*Vh*_*), or FadD from *S. meliloti* 1021 (FadD*_*Sm*_*). After acylation with non-radioactive palmitate, proteins were separated by conformation-sensitive 5 M urea-PAGE and stained with Coomassie blue **(A)**, whereas proteins acylated with radioactive [^3^H]palmitate were separated by 5 M urea-PAGE, transferred to a nitrocellulose membrane, treated with EN^3^HANCE liquid autoradiography enhancer (Perkin Elmer), and the autoradiogram was developed **(B)**. Reactions were performed at 37°C in a water bath for 60 min, as described in section “Materials and methods.” In the assay, 1 mM Triton X-100, 40 μM holo-ACP (holo-ACP CC_1163 or holo-AcpP CC_1677) and 300 nM of each enzyme (AasR*_*Cc*_*, Aas*_*Vh*_*, or FadD*_*Sm*_*) were used. Different isoforms of the ACPs are apo-, holo-, acyl-ACP and ACP dimers. Asterisks indicate bands corresponding to acyl-ACPs, after acylation with non-radioactive palmitate **(A)** or [^3^H]palmitate **(B)**. A typical experiment of three replicates is shown.

In order to confirm that acyl residues are covalently linked to the respective ACPs from *C. crescentus*, acylation assays with elevated enzyme concentrations of AasR*_*Cc*_*, Aas*_*Vh*_*, or FadD*_*Sm*_* were employed to achieve near complete conversion of holo-ACPs to acyl-ACPs. Potentially acylated ACP preparations were separated by conformation-sensitive 5 M urea-PAGE. Gels containing samples obtained with non-radioactive palmitate were stained with Coomassie blue (Figure 4A). Potential acyl-ACP samples generated by using [^3^H]palmitate were separated by 5 M urea-PAGE and autoradiograms were obtained (Figure 4B).

In acylation assays involving non-radioactive palmitate, both ACPs AcpR*_*Cc*_* and AcpP*_*Cc*_* changed their migration in the conformation-sensitive urea-PAGE. Acyl-AcpR*_*Cc*_* migrated slightly slower than the holo-form (between the apo- and the holo-forms) after being incubated with the AasR*_*Cc*_* from *C. crescentus* (Figure 4A), which corresponds to the band that had incorporated [^3^H]palmitate in the radiolabel assay (Figure 4B). When holo-AcpR*_*Cc*_* was treated with Aas from *V. harveyi* or with FadD from *S. meliloti* the slight shift in migration of AcpR*_*Cc*_* did not occur (Figure 4A) nor was any radioactive palmitate attached to AcpR*_*Cc*_* (Figure 4B). This result confirms that the putative ACS is really an acyl-ACP synthetase that can covalently link a fatty acyl residue to the specialized AcpR*_*Cc*_* for sphingolipid biosynthesis in *C. crescentus*. Incubation of AcpP*_*Cc*_* with Aas from *V. harveyi* led to the formation of a protein band that migrated as expected for an acyl-AcpP*_*Cc*_* and much faster than the unacylated AcpP*_*Cc*_* (Figure 4A). Also, the fast-migrating band had incorporated radiolabeled palmitate (Figure 4B), confirming that this substance is acyl-AcpP*_*Cc*_*. However, when AcpP*_*Cc*_* was treated with AasR*_*Cc*_* from *C. crescentus* or with FadD from *S. meliloti*, the strong shift in migration of AcpP*_*Cc*_* did not occur (Figure 4A) nor was any palmitate attached to AcpP*_*Cc*_* in radiolabel assays (Figure 4B). These results show that Aas from *V. harveyi* is an efficient acylation tool for AcpP, but not for AcpR.

**FIGURE 5:**
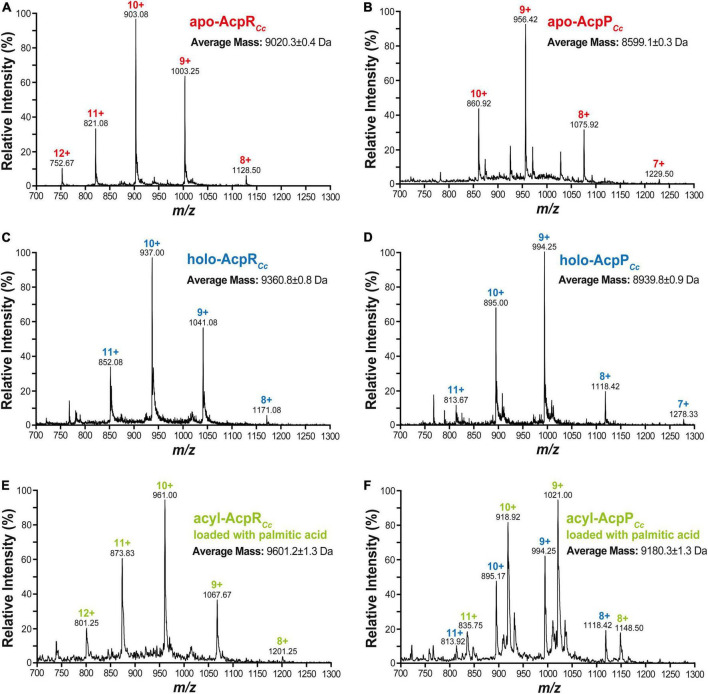
Mass spectrometric analyses of caulobacterial acyl carrier protein preparations. Caulobacterial acyl carrier proteins AcpR*_*Cc*_* or AcpP*_*Cc*_* were prepared as apo- **(A,B)**, holo- **(C,D)**, and acylated (palmitoylated) **(E,F)** versions and their identities were confirmed by HPLC/ESI/MS. In the spectra for each protein sample, multiple-charged species are highlighted with the respective *m/z* values and the corresponding charge; these data were used to calculate the average mass of each protein.

Mass spectrometric analyses of caulobacterial ACPs revealed an average mass of 9,020.3 ± 0.4 Da for AcpR (Figure 5A), which is close to the theoretical molecular weight of 9,022.4 Da calculated from the amino acid sequence for the apo-form of AcpR using the ExPASy – SIB Bioinformatics Resource Portal.^1^ Similarly, an average mass of 8,599.1 ± 0.3 Da was determined for the purified AcpP preparation (Figure 5B). However, the theoretical molecular weight calculated from the complete amino acid sequence amounted to 8,732.4 Da. In proteins where the second amino acid residue is a serine, the N-terminal methionine residue is usually removed by methionine aminopeptidase (Frottin et al., 2006) to generate mature proteins. This post-translational methionine removal is frequently found for housekeeping AcpPs (Platt et al., 1990). An apo-AcpP that lacks the N-terminal methionine (mass 131.2 Da) would have a theoretical mass of 8,601.7 Da, which is in close agreement with the determined average mass of 8,599.1 ± 0.3 Da (Figure 5B). Treatment of apo-ACPs with holo-ACP synthase AcpS leads to species with an average mass of 9,360.8 ± 0.8 Da in the case of AcpR (Figure 5C) or of 8,939.8 ± 0.9 in the case of AcpP (Figure 5D), corresponding to mass increases of 340.5 or 340.7, respectively, when compared to the apo-forms, and as would be expected for the addition of a 4'-PPT group (mass 339). Treatment of holo-AcpR with AasR in the presence of palmitate and ATP led to a species with an average mass of 9,601.2 ± 1.3 Da (Figure 5E) and the observed mass increase of 240.4 is in agreement with the attachment of a palmitoyl residue (+238) to the 4'-PPT arm of AcpR. When holo-AcpP was treated with Aas*_*Vh*_* in the presence of palmitate and ATP a species with an average mass of 9,180.3 ± 1.3 Da was formed (Figure 5F) and the observed mass increase of 240.5 is in agreement with an attachment of a palmitoyl residue to the 4'-PPT arm of AcpP. However, in this latter case, conversion of holo-AcpP to palmitoyl-AcpP was not complete (Figure 5F), probably due to the fact that the acylation reaction was performed in the absence of Triton X-100 to avoid serious contaminations of the mass spectrometer. Theoretical and measured masses as well as the relative errors are given for the distinct caulobacterial ACP versions (Supplementary Table 1). In summary, only acyl-ACP synthetase from *V. harveyi* is able to acylate the housekeeping AcpP and only AasR can efficiently acylate the specialized AcpR from *C. crescentus*.

### Enzymological properties of acyl-ACP synthetase AasR

In order to measure AasR activity in a reproducible way, we investigated the influence of some components on the enzyme assay. The effect of the non-ionic detergent Triton X-100 concentration on the standard AasR assay was studied (Supplementary Figure 6), demonstrating that under these conditions there was a strong detergent dependence for the AasR activity with a maximal activity at 1 mM (0.06%; w/v) Triton X-100. At higher concentrations, Triton X-100 quickly became inhibitory, indicating that substrate dilution kinetics was followed (Eaton and Dennis, 1976).

**FIGURE 6:**
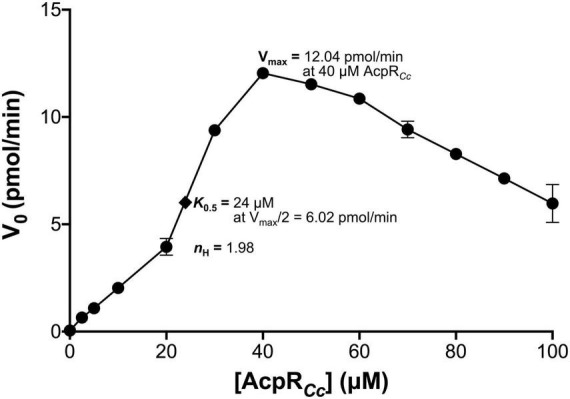
Dependence of AasR activity on holo-AcpR. Holo-AcpR (CC_1163) acylation with AasR (CC_1165) from *C. crescentus* was assayed using [^3^H]palmitate and different concentrations of the holo-AcpR (CC_1163) substrate. Activity was measured by analyzing the incorporation of [^3^H]palmitate into palmitoyl-AcpR product, as described in the Materials and Methods. In the assay, 1 mM Triton X-100 and 75 nM of AasR (CC_1165) were used. Standard deviations of three replicates are shown.

Also, the effect of the AcpR substrate concentration on AasR activity was studied (Figure 6). Clearly, the initial velocity V_0_ for the AasR-catalyzed reaction does not follow a Michaelis-Menten-like hyperbolic dependence on AcpR substrate concentration. In contrast, AasR exhibited a sigmoidal AcpR activation curve, with a measured maximum velocity (V_max_ = 12 pmol/min) at 40 μM of AcpR. The constant, where half of V_*max*_ is obtained (K_0.5_ = 24 μM), indicates a high affinity of AasR for its AcpR substrate. A Hill coefficient of *n*_*H*_ = 1.98 indicates positive cooperativity for AcpR binding to the AasR. Surprisingly, higher concentrations of AcpR (>40 μM) inhibit the AasR-catalyzed reaction (Figure 6).

### Rhodobacterial SPTs require specialized AcpRs for *in vivo* 3-oxo-sphinganine formation

**FIGURE 7:**
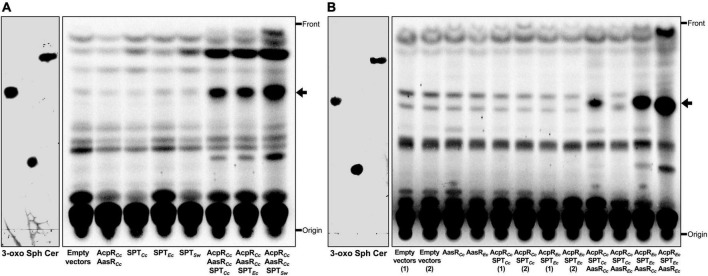
Substrate specificities in the *Rhodobacteria* tripartite protein system for 3-oxo-sphinganine formation. Expression of the structural genes for AcpR*_*Cc*_* and AasR*_*Cc*_* from *C. crescentus* in combination with any of three structural genes for SPT, from *C. crescentus* (SPT*_*Cc*_*), *E. coli* BL21(DE3) (SPT*_*Ec*_*), or *S. wittichii* (SPT*_*Sw*_*) in *E. coli* leads to the formation of a compound that migrates like 3-oxo-sphinganine in TLC **(A)** and AasR*_*Cc*_* from *C. crescentus* displays a broader substrate specificity than AasR*_*Ec*_* from *E. coli* BL21(DE3) **(B)**. Radiolabeling with ^14^C-acetate was performed on complex medium at 30°C for 4 h (transition of exponential to stationary phase of growth) after induction with IPTG at an OD_600_ = 0.3 with *E. coli* BL21(DE3) × pLysS expressing different sphingolipid biosynthesis genes, essentially as described previously (Olea-Ozuna et al., 2021). Strains of *E. coli* BL21(DE3) × pLysS employed harbored **(A)** the empty vectors pCDFDuet-1 and pET9a (Empty vectors), pJPG14 and pET9a (AcpR*_*Cc*_* AasR*_*Cc*_*), pCDFDuet-1 and pJPG08 (SPT*_*Cc*_*), pCDFDuet-1 and pJPG01 (SPT*_*Ec*_*), pCDFDuet-1 and pJPG02 (SPT*_*Sw*_*), pJPG14 and pJPG08 (AcpR*_*Cc*_* AasR*_*Cc*_* SPT*_*Cc*_*), pJPG14 and pJPG01 (AcpR*_*Cc*_* AasR*_*Cc*_* SPT*_*Ec*_*), and pJPG14 and pJPG02 (AcpR*_*Cc*_* AasR*_*Cc*_* SPT*_*Sw*_*). In panel **(B)** *E. coli* BL21(DE3) × pLysS harbored the empty vectors pCDFDuet-1 and pET16b [Empty vectors (1)], the empty vectors pCDFDuet-1 and pET17b [Empty vectors (2)], pCDFDuet-1 and pDG10 (AasR*_*Cc*_*), pCDFDuet-1 and pJPG17 (AasR*_*Ec*_*), pJPG16 and pET16b [AcpR*_*Cc*_* SPT*_*Cc*_* (1)], pJPG16 and pET17b [AcpR*_*Cc*_* SPT*_*Cc*_* (2)], pJPG20 and pET16b [AcpR*_*Ec*_* SPT*_*Ec*_* (1)], pJPG20 and pET17b [AcpR*_*Ec*_* SPT*_*Ec*_* (2)], pJPG16 and pDG10 (AcpR*_*Cc*_* SPT*_*Cc*_* AasR*_*Cc*_*), pJPG16 and pJPG17 (AcpR*_*Cc*_* SPT*_*Cc*_* AasR*_*Ec*_*), pJPG20 and pDG10 (AcpR*_*Ec*_* SPT*_*Ec*_* AasR*_*Cc*_*), and pJPG20 and pJPG17 (AcpR*_*Ec*_* SPT*_*Ec*_* AasR*_*Ec*_*). At the end of the labeling period, cells were harvested, lipids were extracted, separated by TLC and developed chromatograms were subjected to autoradiography as previously described (Olea-Ozuna et al., 2021). The arrow indicates a compound migrating like 3-oxo-sphinganine. Reference compounds 3-oxo-sphinganine (3-oxo), sphinganine (Sph), and *N*-palmitoyl-D-sphingosine (Cer) were developed in the same TLC and visualized by iodine staining.

The lipid profiles of *E. coli* strains expressing individual genes or combinations of genes required for 3-oxo-sphinganine synthesis were analyzed by TLC. If the combination of AcpR*_*Cc*_* and AasR*_*Cc*_* or individual SPTs (SPT*_*Cc*_*, SPT*_*Ec*_*, or SPT*_*Sw*_*) were expressed, the lipid profile of those samples was similar to the profile obtained from an *E. coli* strain harboring empty vectors pET9a and pCDFDuet-1 (Figure 7A). We had previously shown that the combined expression of AcpR*_*Cc*_*, AasR*_*Cc*_* and SPT*_*Cc*_* in *E. coli* led to the formation of a compound that migrated like 3-oxo-sphinganine in TLC analyses and high resolution mass spectrometry analysis of such lipid extracts indicated the presence of a compound with *m/z* = 300.28953 expected for C18-3-oxo-sphinganine (Olea-Ozuna et al., 2021). We now demonstrate that the combined expression of AcpR*_*Cc*_*/AasR*_*Cc*_*, together with any of the three SPTs (SPT*_*Cc*_*, SPT*_*Ec*_*, or SPT*_*Sw*_*) studied, led to abundant formation of compounds that migrated like the 3-oxo-sphinganine standard (Figure 7A). Also, in the cases of all three SPTs, compounds that migrated like sphinganine or ceramide were formed, possibly by intrinsic enzymes of *E. coli* BL21(DE3) (Supplementary Figure 7). The gene ECD_02852 might encode a keto-sphinganine reductase and convert 3-oxo-sphinganine to sphinganine and the gene product of ECD_02850 might be an *N*-acyltransferase that converts sphinganine to (dihydro)ceramide. Surprisingly, all newly formed compounds were more intense when the previously characterized SPT from *S. wittichii* (Raman et al., 2010) was employed (Figure 7A). These results suggest that the acylated AcpR*_*Cc*_* serves as an excellent substrate for each of the three SPTs investigated.

### Intrinsic AcpR and AasR are needed for efficient 3-oxo-sphinganine formation in *Caulobacter*

To further clarify the requirement of ACPs for 3-oxo-sphinganine formation, housekeeping and specialized ACPs from *C. crescentus* and *E. coli* BL21, respectively, were coexpressed with acyl-ACP synthetase (AasR*_*Cc*_*) and serine palmitoyltransferase (SPT*_*Cc*_*) from *C. crescentus*. 3-oxo-sphinganine formation was observed when AcpR*_*Cc*_* was coexpressed (Supplementary Figure 8A), but not upon coexpression of any of the housekeeping ACPs (AcpP*_*Cc*_* or AcpP*_*Ec*_*). When AcpR*_*Ec*_* from *E. coli* was coexpressed, a compound migrating like 3-oxo-sphinganine was also formed, but to a lesser extent than upon expression of AcpR*_*Cc*_* (Supplementary Figure 8A). Therefore, AasR*_*Cc*_* can acylate both, its native AcpR*_*Cc*_* and to a lesser extent AcpR*_*Ec*_* from *E. coli*. Therefore, in this tripartite system (AasR*_*Cc*_* AcpR*_*Cc*_* SPT*_*Cc*_*) the specific AcpR*_*Cc*_* from *C. crescentus* is required for efficient 3-oxo-sphinganine formation. Also, when we studied coexpression of distinct acyl-CoA/acyl-ACP synthetases (AasR*_*Cc*_* Aas*_*Vh*_* FadD*_*Sm*_*) with the specialized ACP (AcpR*_*Cc*_*) and serine palmitoyltransferase (SPT*_*Cc*_*) from *C. crescentus*, 3-oxo-sphinganine was only formed when the specific acyl-ACP synthetase (AasR*_*Cc*_*) from *C. crescentus* was coexpressed (Supplementary Figure 8B), but not upon coexpression of an acyl-ACP synthetase (Aas*_*Vh*_*) from *V. harveyi* or coexpression of an acyl-CoA synthetase (FadD*_*Sm*_*) from *S. meliloti.*

### AasRs require their intrinsic AcpRs for efficient 3-oxo-sphinganine formation *in vivo*

Finally, upon coexpression of cognate acyl-ACP synthetase (AasR*_*Ec*_*), serine palmitoyltransferase (SPT*_*Ec*_*), and specialized ACP (AcpR*_*Ec*_*) from *E. coli*, formation of 3-oxo-sphinganine was observed (Figure 7B). This did not occur when AcpR*_*Cc*_* and SPT*_*Cc*_* from *C. crescentus* were coexpressed with AasR*_*Ec*_* from *E. coli.* However, when AcpR*_*Ec*_* and SPT*_*Ec*_* from *E. coli* were coexpressed with AasR*_*Cc*_* from *C. crescentus* a compound migrating like 3-oxo-sphinganine was formed. These data suggest that AasR*_*Cc*_* from *C. crescentus* can acylate its native AcpR*_*Cc*_* and to a lesser extent AcpR*_*Ec*_* from *E. coli.* In contrast, AasR*_*Ec*_* from *E. coli* can acylate AcpR*_*Ec*_* from *E. coli*, but not AcpR*_*Cc*_* from *C. crescentus*. Therefore, the specific interaction between AasR and AcpR from the same organism seems crucial for acyl-AcpR and efficient 3-oxo-sphinganine formation.

### Serine palmitoyltransferases from *Caulobacter crescentus*, *Escherichia coli* BL21(DE3) and *Sphingomonas wittichii* prefer acyl-AcpR as thioester substrate

To determine which potential thioester substrates (acyl-CoA, acyl-AcpR*_*Cc*_* or acyl-AcpP*_*Cc*_*) were preferred by each SPT, enzymatic assays were performed similar to those described by Ikushiro et al. (2001). Cell-free extracts of *E. coli* BL21(DE3) × pLysS, in which SPT from *C. crescentus*, *E. coli* BL21(DE3) or *S. wittichii* (Raman et al., 2010) had been overexpressed, were used to study 3-oxo-sphinganine formation in the presence of L-[^14^C]serine and one of the potential thioester substrates.

**FIGURE 8:**
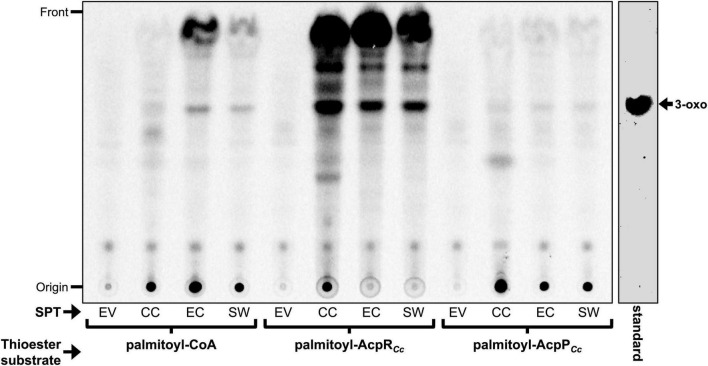
Dependence of serine palmitoyltransferases (SPTs) from different *Rhodobacteria* on distinct acyl-thioester substrates. Analysis of the incorporated radioactivity (L-[^14^C]serine) in lipid products after enzymatic assays using CE of *E. coli* BL21(DE3) × pLysS with empty vector pET9a (EV), CE with SPT (CC_1162) of *C. crescentus* (CC), SPT (ECD_02854) of *E. coli* (EC), or SPT (Swit_3900) of *S. wittichii* (SW). The respective thioester substrates [palmitoyl-CoA, palmitoyl-AcpR*_*Cc*_* (CC_1163) and palmitoyl-AcpP*_*Cc*_* (CC_1677)] used in the reaction are indicated. Iodine stained 3-oxo-sphinganine (3-oxo) standard (Matreya LLC) is shown.

The *in vitro* formation of a minor compound migrating like 3-oxo-sphinganine in TLC was observed when using the SPTs from *C. crescentus*, *E. coli* BL21(DE3) or *S. wittichii*, and palmitoyl-CoA as thioester substrate (Figure 8). However, no formation of 3-oxo-sphinganine was observed when using cell-free *E. coli* extracts harboring the empty plasmid pET9a. When palmitoyl-AcpR*_*Cc*_* was employed as thioester substrate, several lipidic compounds, including a major compound migrating like 3-oxo-sphinganine were formed by SPTs from *C. crescentus*, *E. coli* BL21(DE3), or *S. wittichii* but none in the absence of any SPT (EV) (Figure 8). Presently, we do not know the chemical nature of the other compounds formed in an SPT-dependent way. However, as 3-oxo-sphinganine possesses both functional groups, a primary amine and an oxo group, required for abiotic, chemical imine (Schiff base) formation, it is likely that 3-oxo-sphinganine undergoes condensations by Schiff reactions with itself and other compounds carrying oxo- or primary amine groups. In fact, the fastest migrating compound in Figure 8 is also formed from a 3-oxo-sphinganine standard when incubated with buffer only. When palmitoyl-AcpP was used as thioester substrate, only minor amounts of a compound that migrated like 3-oxo-sphinganine were formed by all three SPTs, suggesting that palmitoyl-AcpP*_*Cc*_* is not a good substrate for any of these three rhodobacterial SPTs (Figure 8). Therefore, for all three SPTs from the *Rhodobacteria* assayed [*C. crescentus*, *E. coli* BL21(DE3), and *S. wittichii*], palmitoyl-AcpR*_*Cc*_* was much preferred as thioester substrate over the acylated AcpP*_*Cc*_* or acyl-CoA derivatives.

### Relaxed substrate specificity of serine palmitoyltransferases from *Rhodobacteria*

Kinetic studies with purified SPTs from *S. paucimobilis* and *S. wittichii* suggest that the affinities for the substrates serine (K_*m*_ 1 mM) and palmitoyl-CoA (K_*m*_ 30 μM) are similar for both enzymes (Raman et al., 2010). However, turnover numbers and catalytic efficiencies of the *S. paucimobilis* SPT were found to be at least one order of magnitude higher than those for the *S. wittichii* SPT (Raman et al., 2010). Based on these results, the authors suggested that a specialized acyl-AcpR might be a better substrate for *S. wittichii* SPT than acyl-CoA and that this might not be the case for the well-characterized *S. paucimobilis* SPT. Our results (Figure 8) show that even the acyl-AcpR from *C. crescentus* is a better substrate for SPT from *S. wittichii* than acyl-CoA. However, we cannot confirm the assumption that *S. paucimobilis* SPT is not accompanied by a cognate AcpR (Raman et al., 2010) encoded in its genome. Our data base analysis of available *S. wittichii* and *S. paucimobilis* genomes (Supplementary Table 2) suggests that essentially all of them possess structural genes for AcpR and AasR and that therefore an AasR/AcpR-dependent SPT-catalyzed reaction occurs in the *Sphingomonas* genus.

In this work, we demonstrated that the specialized AcpR for sphingolipid biosynthesis from *C. crescentus* was the preferred thioester substrate (acyl-AcpR) for the SPTs from *C. crescentus* CB15, *E. coli* BL21(DE3), and *S. wittichii* to form the sphingolipid precursor, 3-oxo-sphinganine (Figure 7). SPTs from these bacteria were also able to use acyl-CoA or the housekeeping acyl-AcpP from *C. crescentus* as thioester substrate, however, to a much lesser extent. Therefore, although all three SPTs from *Rhodobacteria* showed a relaxed thioester substrate specificity, they clearly preferred the specialized ACP AcpR for initiating sphingolipid biosynthesis.

### Genomic organization of rhodobacterial sphingolipid biosynthesis genes

**FIGURE 9:**
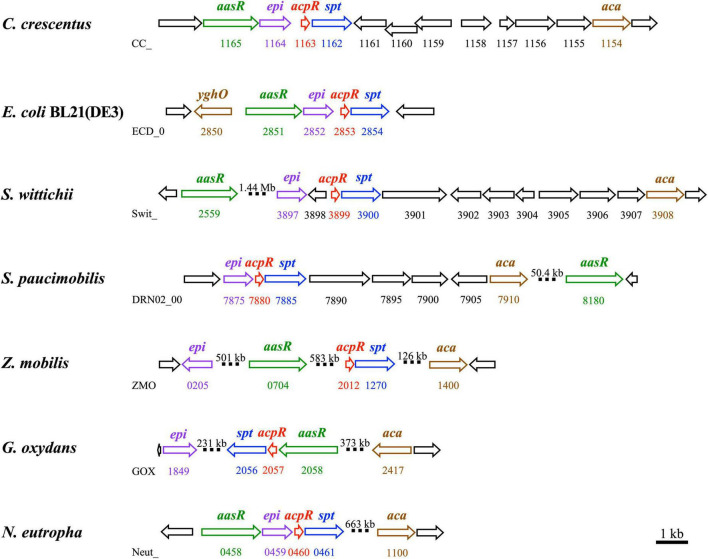
Genomic organization of genes/operons for sphingolipid biosynthesis in *Rhodobacteria*. Operons containing genes involved in the biosynthesis of sphingolipids from *Caulobacter crescentus* CB15, *Escherichia coli* BL21(DE3), *Sphingomonas wittichii* RW1, *Sphingomonas paucimobilis* strain AIMST S2, *Zymomonas mobilis* ZM4, *Gluconobacter oxydans* 621H, and *Nitrosomonas eutropha* C91 are shown. Three genes (*aasR*: CC_1165, ECD_02851, Swit_2559, DRN02_008180, ZMO0704, GOX2058, and Neut_0458; *acpR*: CC_1163, ECD_02853, Swit_3899, DRN02_007880, ZMO2012, GOX2057, and Neut_0460; and *spt*: CC_1162, ECD_02854, Swit_3900, DRN02_007885, ZMO1270, GOX2056, and Neut_0461) from each strain, respectively, and required for the biosynthesis of the sphingolipid precursor, 3-oxo-sphinganine, are highlighted. Also, two genes (*epi*: CC_1164, ECD_02852, Swit_3897, DRN02_007875, ZMO0205, GOX1849, and Neut_0459; and *aca*: CC_1154, *yghO* ECD_02850, Swit_3908, DRN02_007910, ZMO1400, GOX2417, and Neut_1100) from each strain, respectively, and required for dihydroceramide biosynthesis are shown. Structural genes for specialized acyl-ACP synthetase (*aasR*), specialized acyl carrier protein (*acpR*), serine palmitoyltransferase (*spt*), predicted dehydrogenase/epimerase (*epi*), and acyl-CoA *N*-acyltransferase (*aca*) are highlighted with colors (green, red, blue, violet, and brown, respectively). Large genomic distances are indicated by interrupted bars and indication of the respective distance is shown. Accession numbers for the genes mentioned above are listed in the legend of Supplementary Table 2.

In eukaryotes the first committed step of sphingolipid biosynthesis is catalyzed by SPT and consists in the decarboxylative condensation of serine with an acyl-CoA, resulting in the formation of 3-oxo-sphinganine. Although palmitoyl-CoA can serve as a substrate for SPT from *C. crescentus* (Stankeviciute et al., 2022), we have now demonstrated for *C. crescentus* that a fatty acyl residue is linked to the specialized ACP AcpR by the specialized acyl-ACP synthetase AasR, and that subsequently acyl-AcpR is used as the preferred acyl-thioester substrate in the SPT-catalyzed reaction (Figure 1). Also, our *in vivo* experiments indicate that AcpR/AasR pairs from the same organism together with SPT are required for efficient 3-oxo-sphinganine formation (Figure 7 and Supplementary Figure 8). Therefore, the biological significance of the AcpR/AasR proteins might consist of sequestering acyl pools and specifically directing them toward sphingolipid synthesis. This intricate formation of 3-oxo-sphinganine seems to be common in members of the *Rhodobacteria* as their structural gene for SPT is preceded by a structural gene for the specialized AcpR, both forming an operon (Figure 9 and Supplementary Table 2). Also, a structural gene for the specialized acyl-ACP synthetase (*aasR*) is found in genomes of other sphingolipid-producing *Rhodobacteria* (Figure 9 and Supplementary Table 2). Whereas in most *Rhodobacteria aasR* is located in the close neighborhood of the *acpR-spt* operon, in the *Sphingomonadaceae* (*Sphingomonas*, *Zymomonas*) *aasR* is encountered in distant parts of the genome (Figure 9 and Supplementary Table 2). It is thought that also in bacteria keto-sphinganine reductase would covert oxo-sphinganine to sphinganine which would subsequently be acylated by an acyl-CoA *N*-acyltransferase during dihydroceramide biosynthesis (Olea-Ozuna et al., 2021). Recently, an alternative pathway for ceramide biosynthesis in *C. crescentus* was proposed in which *N*-acylation would precede reduction (Stankeviciute et al., 2022). Good homologs are encountered for the presumptive keto-sphinganine reductase (*epi*) and acyl-CoA *N*-acyltransferase (aca) (Olea-Ozuna et al., 2021) as well in other SPT-containing *Rhodobacteria* (Figure 9 and Supplementary Table 2). These five structural genes for dihydroceramide synthesis in *Rhodobacteria* are clustered together only in the genomes of *C. crescentus* and *E. coli* BL21 (Figure 9 and Supplementary Table 2).

### Specialized acyl carrier proteins as substrates for rhodobacterial SPTs

**FIGURE 10:**
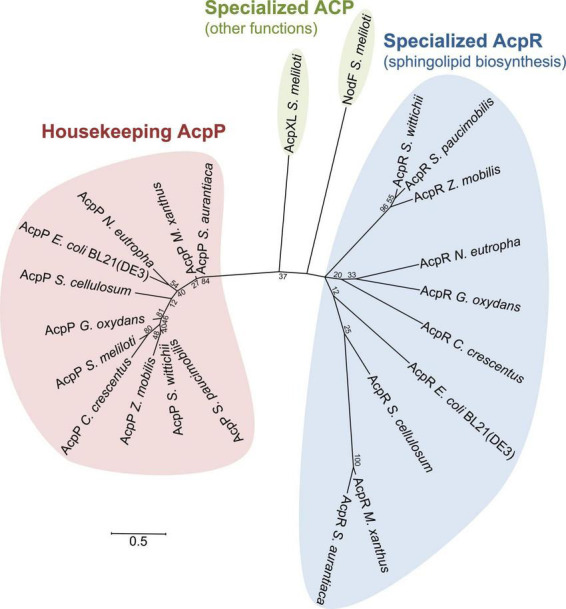
Unrooted phylogenetic tree of selected ACPs, including housekeeping AcpPs and AcpRs for sphingolipid biosynthesis in *Rhodobacteria*. The amino acid sequences were aligned using the CLUSTAL OMEGA program (https://www.ebi.ac.uk/Tools/msa/clustalo/). The gap opening and extension parameters were set to 10 and 0.1, respectively. The tree was constructed using the program MEGA version X (http://www.megasoftware.net/) employing the maximum likelihood method. Distances between sequences are expressed as 0.5 changes per amino acid residue. The number at each node represents the bootstrap value as a percentage of 100 replications. ORF names/accession numbers are as follows: *Caulobacter crescentus* CB15 (AcpR: CC_1163/AAK23147.1; AcpP: CC_1677/AAK23655.1), *Escherichia coli* BL21(DE3) (AcpR: ECD_02853/ACT44657.1; AcpP: ECD_01090/ACT42985.1), *Gluconobacter oxydans* 621H (AcpR: GOX2057/AAW61793.1; AcpP: GOX2041/AAW61777.1), *Myxococcus xanthus* DK 1622 (AcpR: MXAN_6637/ABF91660.1; AcpP: MXAN_4769/ABF90332.1), *Nitrosomonas eutropha* C91 (AcpR: Neut_0460/ABI58737.1; AcpP: Neut_0467/ABI58744.1), *Sinorhizobium meliloti* 1021 (NodF: SMa0852/AAK65123.1; AcpXL: SMc04278/CAC46521.1; AcpP: SMc00573/CAC45722.1), *Sorangium cellulosum* So ce56 (AcpR: sce7052/CAN97221.1; AcpP: sce3814/CAN93974.1), *Sphingomonas paucimobilis* (AcpR: DRN02_007880/QBE91941.1; AcpP: WP_007689016.1), *Sphingomonas wittichii* RW1 (AcpR: Swit_3899/ABQ70244.1; AcpP: Swit_0088/ABQ66460.1), *Stigmatella aurantiaca* DW4/3-1 (AcpR: STAUR_1257/ADO69061.1; AcpP: STAUR_5619/ADO73384.1), and *Zymomonas mobilis* ZM4 (AcpR: ZMO2012/ADK75091.1; AcpP: ZMO1279/AAV89903.1).

All bacteria possess an essential housekeeping ACP, which is expressed constitutively from the *acpP* gene (Rawlings and Cronan, 1992). Phylogenetic analysis of amino acid sequences from housekeeping (AcpP) and specialized acyl carrier proteins showed that they cluster separately (Figure 10). Housekeeping AcpPs from α- (*C. crescentus*, *S. wittichii*, *S. paucimobilis*, *Z. mobilis*, *G. oxydans*, and *S. meliloti*), β- (*N. eutropha*), γ- [*E. coli* BL21(DE3)], and δ- (*S. cellulosum*, *M. xanthus*, and *S. aurantiaca*) *Proteobacteria* group together. Also, specialized ACPs for sphingolipid biosynthesis (AcpRs) cluster together but clearly form a distinct subgroup of ACPs.

Notably, besides the housekeeping AcpP, distant AcpR homologs are also encoded in genomes of the δ-proteobacteria *Sorangium cellulosum*, *Myxococcus xanthus*, and *Stigmatella aurantiaca*. Other specialized ACPs with other functions, i.e., NodF or AcpXL from *S. meliloti*, group separately from both the AcpP and AcpR clusters. It is remarkable that the housekeeping AcpPs cluster more closely together than the specialized AcpRs (Figure 10). The tertiary structure of ACPs seems to be conserved consisting of a 3- or 4-helix bundle comprised of three major α-helices (helices I, II, and IV) and a short α-helical segment (helix III) connecting helices II and IV (Byers and Gong, 2007). For interaction with most enzymes, helix II of AcpP seems to play a dominant role and has been termed “recognition helix” (Zhang et al., 2003). It is noteworthy that most of the acidic amino acyl residues of the C-terminal part of helix II, which are essential for the interaction of AcpP with enzymes, are conserved in AcpRs as well (Supplementary Figure 9).

### Acyl-CoA and acyl-ACP synthetases

**FIGURE 11:**
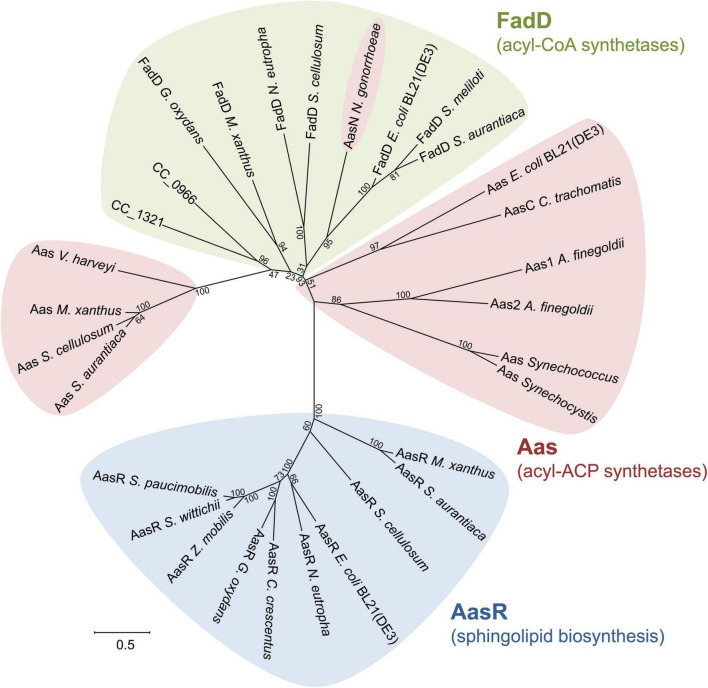
Unrooted phylogenetic tree of selected and putative acyl-CoA/ACP synthetases. Amino acid sequences of distinct acyl-CoA synthetases (FadD), known acyl-ACP synthetases (Aas) and specialized acyl-ACP synthetases (AasR) for sphingolipid biosynthesis in *Rhodobacteria* were aligned using the CLUSTAL OMEGA program (https://www.ebi.ac.uk/Tools/msa/clustalo/). The gap opening and extension parameters were set to 10 and 0.1, respectively. The tree was constructed using the program MEGA version X (http://www.megasoftware.net/) employing the maximum likelihood method. Distances between sequences are expressed as 0.5 changes per amino acid residue. The number at each node represents the bootstrap value as a percentage of 100 replications. ORF names/accession numbers are as follows: *Alistipes finegoldii* (Aas1: Alfi_2635/AFL78895; Aas2: Alfi_0371/AFL76767), *Caulobacter crescentus* CB15 (AasR: CC_1165/AAK23149.1; potential FadDs: CC_0966/AAK22950.1, CC_1321/AAK23302.1), *Chlamydia trachomatis* (AasC: CT_776/NP_220295), *Escherichia coli* BL21(DE3) (Aas: ECD_02684/ACT44500.1; AasR: ECD_02851/ACT44655.1; FadD: ECD_01775/ACT43629.1), *Gluconobacter oxydans* 621H (AasR: GOX2058/AAW61794.1; FadD: WP_062447138.1), *Myxococcus xanthus* DK 1622 (Aas: MXAN_6374/ABF88458; AasR: MXAN_6636/ABF92798; FadD: MXAN_7148/ABF89596), *Neisseria gonorrhoeae* (AasN: NGO_0530/AAW89268), *Nitrosomonas eutropha* C91 (AasR: Neut_0458/ABI58735.1; FadD: Neut_1417/ABI59664.1), *Sinorhizobium meliloti* 1021 (FadD: SMc02162/CAC41921.1), *Sorangium cellulosum* So ce56 (Aas: sce5736/CAN95899.1; AasR: sce7053/CAN97222.1; FadD: sce3825/CAN93985.1), *Sphingomonas paucimobilis* strain AIMST S2 (AasR: DRN02_008180/QBE91994.1), *Sphingomonas wittichii* RW1 (AasR: Swit_2559/ABQ68917.1), *Stigmatella aurantiaca* DW4/3-1 (Aas: STAUR_2914/ADO70706; AasR: STAUR_1258/ADO69062; FadD: STAUR_3279/ADO71071), *Synechococcus elongatus* PCC 7942 (Aas: Synpcc7942_0918/ABB56948), *Synechocystis* sp. PCC 6803 (Aas: slr1609/BAA17024), *Vibrio harveyi* (Aas: LA59_RS23465/WP_017819392.1), and *Zymomonas mobilis* subsp. *mobilis* ZM4 (AasR: ZMO0704/AAV89328.2).

Phylogenetic analysis of selected fatty acyl AMP ligases clarifies that at least three subgroup clusters exist (Figure 11) consisting of FadD-like acyl-CoA synthetases, acyl-AcpP synthetases employing the housekeeping AcpP as thiol substrate, and AasR-like acyl-AcpR synthetases required for sphingolipid biosynthesis in *Rhodobacteria*. Although there is experimental evidence for acyl-CoA synthetase activity for the annotated ORFs in *S. meliloti* (Soto et al., 2002) and *E. coli*, FadD candidates for *G. oxydans* and *N. eutropha* are assigned based on similarity to the *E. coli* and *S. meliloti* FadD (Supplementary Table 3). *C. crescentus* can grow on oleate as sole carbon source, possesses acyl-CoA synthetase activity and degrades oleate by β-oxidation (O’Connell et al., 1986). P-BLAST searches with FadD from *E. coli* or *S. meliloti* identify 11 candidates for acyl-CoA synthetases/fatty-acid CoA ligases in the *C. crescentus* genome and the two best candidates for acyl-CoA synthetase, CC_0966 and CC_1321 (Supplementary Table 3), are included in Figure 11. However, if free fatty acids are activated to their CoA derivatives, one might expect that they are catabolized by β-oxidation. In contrast, if free fatty acids are converted to acyl-AcpR derivatives, also at the energetic cost of 2 ATP molecules (Figure 1), they are redirected to sphingolipid biosynthesis.

Although the biological significance of acyl-ACP synthetases is not always clear yet, acyl-ACP synthetase of *E. coli* together with 1-acylglycerol phosphate acyltransferase PlsC is a key player in reacylating lysophospholipids, for example in the lyso-phosphatidylethanolamine cycle (Sahonero-Canavesi et al., 2019). Similarly, a major function of acyl-ACP synthetases in the cyanobacteria *Synechocystis* and *Synechococcus* seems to consist in the recycling of endogenous fatty acids (Kaczmarzyk and Fulda, 2010). In contrast, the intracellular parasite *Chlamydia trachomatis* (Yao et al., 2015), pathogenic *Neisseria* (Yao et al., 2016), or the Bacteroidete *Alistipes* (Radka et al., 2020) from the human gut microbiome use their acyl-ACP synthetases to scavenge host fatty acids in order to incorporate them into their membrane lipids. Acyl-ACP synthetases acylating the housekeeping AcpP form at least two subgroups, one containing Aas from *E. coli* and another containing Aas from *V. harveyi*, which are distinct from acyl-CoA synthetases. In addition, the acyl-ACP synthetase of *Neisseria* clusters together with acyl-CoA synthetases, suggesting that acyl-ACP synthetases acylating housekeeping AcpPs might have evolved on several occasions from acyl-CoA synthetases.

Members of a third cluster involving possible specialized Aas for sphingolipid biosynthesis in *Rhodobacteria* (AasR), i.e., from the α- (*C. crescentus*, *S. wittichii*, *S. paucimobilis*, *G. oxydans*, and *Z. mobilis*), β- (*N. eutropha*), and γ- [*E. coli* BL21(DE3)] *Proteobacteria* group together, although they seem to form two subgroups (Figure 11). In one subgroup, AasRs from *S. wittichii*, *S. paucimobilis*, and *Z. mobilis* cluster closely together. In the other subgroup, AasRs from *C. crescentus, G. oxydans*, *N. eutropha*, and *E. coli* BL21(DE3) are more distant. Specialized AcpRs from these same bacteria cluster in a similar fashion, forming these same two subgroups (Figure 10). The observed clustering profiles of AcpRs and AasRs are complementary and might indicate a high specificity of these acyl-AcpR synthetases to acylate the specialized AcpRs for sphingolipid biosynthesis in these *Rhodobacteria*. Our own studies (Figure 7) confirm that AcpRs are acylated exclusively or at least preferentially by the AasR from the same organism. Phylogenetic trees of the *Proteobacteria* suggest that early during their diversification, the δ-*Proteobacteria* separated from the others (Madigan et al., 2018). In this context it is remarkable that in addition to homologs of FadD*_*Ec*_* and Aas*_*Vh*_*, the δ-proteobacteria *M. xanthus*, *S. aurantiaca*, and *S. cellulosum* possess good and distinct homologs of AasR*_*Cc*_* (Supplementary Table 4) encoded in their enormous genomes and that these homologs branch off at the base of the AasR-like cluster (Figure 11). Also, *M. xanthus*, *S. aurantiaca*, and *S. cellulosum* have genes coding for AcpR homologs in addition to the structural genes for housekeeping AcpPs (Supplementary Table 4). Notably, *acpR* and *aasR* homologs form an operon in the δ-proteobacteria *M. xanthus*, *S. aurantiaca*, and *S. cellulosum* but are situated in different genomic contexts as they are in the *Rhodobacteria*.

## Conclusion

Sphingolipids are important structural components in membranes of *Eukarya* and participate in numerous cellular functions. In the *Bacteria* domain, sphingolipids occur in members of the phyla *Bacteroidetes* and *Proteobacteria*. During the first step of sphingolipid biosynthesis serine palmitoyltransferase is known to condense acyl-coenzyme A with serine forming 3-oxo-sphinganine as sphingolipid biosynthesis intermediate. In *Rhodobacteria*, which comprise α-, β-, and γ-*Proteobacteria*, the structural gene for serine palmitoyltransferase (*spt*) is usually preceded by a gene predicted to code for an acyl carrier protein (ACP), and a gene predicted to encode an acyl-CoA synthetase. We show that in the α-proteobacterium *Caulobacter crescentus* a specialized acyl carrier protein, AcpR, is selectively acylated by a specialized acyl-ACP synthetase AasR and that acyl-AcpR is the preferred acyl substrate for rhodobacterial serine palmitoyltransferases during 3-oxo-sphinganine formation. This more complex way of 3-oxo-sphinganine synthesis in *Rhodobacteria* might allow them to efficiently channel free fatty acids toward sphingolipid biosynthesis.

## Materials and methods

### Bacterial strains, plasmids and growth conditions

The bacterial strains and plasmids used in this work and their relevant characteristics are listed in Table 1. *E. coli* strains were grown at 30°C either in Luria-Bertani (LB) broth (Sambrook et al., 2001), or in M9 minimal medium (Miller, 1972) for β-[^3^H]alanine labeling assays. *C. crescentus* CB15 strain was cultivated at 30°C in PYE (peptone-yeast extract) complex medium (0.2% peptone, 0.1% yeast extract, 1 mM MgSO_4_ and 0.5 mM CaCl_2_) (Hottes et al., 2004). *S. wittichii* RW1 strain was grown at 30°C in nutrient medium (0.5% peptone and 0.3% meat extract) (Halden et al., 2005). Antibiotics were added to media, when required, at the following final concentrations (μg/ml): carbenicillin 100, chloramphenicol 20, kanamycin 50, and spectinomycin 100, for *E. coli* strains.

**TABLE 1:** Bacterial strains and plasmids.

Strain or plasmid	Relevant characteristics	Source or reference
***Escherichia coli*** DH5α BL21(DE3) OG7001 ***Caulobacter*** ***crescentus***	*rec*A1, ϕ80 *lac*ZΔM15, host used for cloning Host used for protein expression *panD* mutant of BL21(DE3), β-alanine auxotroph	Hanahan, 1983 Studier et al., 1990 Epple et al., 1998
CB15 ***Sphingomonas*** ***wittichii***	Wild type strain; ATCC 19089	Poindexter, 1964
RW1 **Plasmids**	Wild type	DSMZ 6014
pET9a	Expression vector, Kn*^R^*	Studier et al., 1990
pET28a	Expression vector, Kn*^R^*	Novagen
pET16b	Expression vector, Cb*^R^*	Novagen
pET17b	Expression vector, Cb*^R^*	Novagen
pLysS	Causes repression of T7 polymerase, Cm*^R^*	Studier et al., 1990
pCDFDuet-1	Vector for coexpression, Sp*^R^*	Novagen
pBAD24	Arabinose-regulated expression vector, Cb*^R^*	Guzman et al., 1995
pAL07	pET9a carrying *acpXL_*Sm*_*	Dávila-Martínez et al., 2010
pMP2301	pET9a carrying *nodF_*Rl*_*	Ritsema et al., 1998
pTB1003	pET9a carrying *rkpF*_*Sm*_ of Rm41	Epple et al., 1998
pTB5079	pET9a carrying *acpP* from *E. coli* DH5α, identical nucleotide sequence as *acpP* ECD_01090*_*Ec*_* from *E. coli* BL21(DE3)	López-Lara and Geiger, 2000
pSBA01	pET16b carrying *acpS_*Sm*_*	Ramos-Vega et al., 2009
pECH6	pET17b carrying *fadD* smc02162*_*Sm*_*	Pech-Canul et al., 2020
pDG01	pET9a carrying CC_1163 (*acpR*_*Cc*_)	Olea-Ozuna et al., 2021
pJPG08	pET9a carrying CC_1162 (*spt*_*Cc*_)	Olea-Ozuna et al., 2021
pRJ03	pET9a carrying CC_1163/CC_1162 (*acpR*_*Cc*_/*spt*_*Cc*_)	Olea-Ozuna et al., 2021
pECH3	pET28a carrying *fadD* smc02162*_*Sm*_*	This study
pDG04	pET17b carrying CC_1165 (*aasR*_*Cc*_)	This study
pDG10	pET16b carrying CC_1165 (*aasR*_*Cc*_)	This study
pPEG01	pET9a carrying CC_1677 (*acpP*_*Cc*_)	This study
pJPG01	pET9a carrying ECD_02854 (*spt*_*Ec*_)	This study
pJPG02	pET9a carrying Swit_3900 (*spt*_*Sw*_)	This study
pJPG05	pBAD24 carrying CC_1162 (*spt*_*Cc*_)	This study
pJPG06	pBAD24 carrying ECD_02854 (*spt*_*Ec*_)	This study
pJPG07	pBAD24 carrying Swit_3900 (*spt*_*Sw*_)	This study
pJPG10	pET16b carrying *aasS* from *V. harveyi*	This study
pJPG12	pET9a carrying ECD_02853 (*acpR*_*Ec*_)	This study
pJPG13	pCDFDuet-1 carrying CC_1165 (*aasR*_*Cc*_) in MCS-1	This study
pJPG14	pCDFDuet-1 carrying CC_1165 (*aasR*_*Cc*_) in MCS-1 and CC_1163 (*acpR*_*Cc*_) in MCS-2	This study
pJPG15	pCDFDuet-1 carrying CC_1165 (*aasR*_*Cc*_) in MCS-1 and CC_1162 (*spt*_*Cc*_) in MCS-2	This study
pJPG16	pCDFDuet-1 carrying CC_1163/CC_1162 (*acpR*_*Cc*_/*spt*_*Cc*_) in MCS-2	This study
pJPG17	pET17b carrying ECD_02851 (*aasR*_*Ec*_)	This study
pJPG20	pCDFDuet-1 carrying ECD_02853/ECD_02854 (*acpR*_*Ec*_/*spt*_*Ec*_) in MCS-2	This study

Kn^R^, Cb^R^, Cm^R^, Sp^R^: kanamycin, carbenicillin, chloramphenicol, spectinomycin resistance, respectively.

Subscripts _Cc_, _Ec_, _Rl_, _Sm_ and _Sw_ denote genes from C. crescentus CB15, E. coli BL21(DE3), R. leguminosarum, S. meliloti 1021 and S. wittichii RW1, respectively.

DSMZ, Deutsche Sammlung von Mikroorganismen and Zellkulturen strain collection.

### DNA manipulations

Recombinant DNA techniques were carried out using standard procedures (Sambrook et al., 2001). Commercial sequencing of amplified genes by Eurofins Medigenomix (Martinsried, Germany) corroborated correct DNA sequences. DNA regions containing presumptive sphingolipid biosynthesis genes and derived protein sequences were analyzed using the National Center for Biotechnology Information (NCBI) BLAST network center (Altschul et al., 1997).

### Construction of expression plasmids

Genes of interest were amplified by PCR from genomic DNA of *C. crescentus* CB15, *E. coli* BL21(DE3), or *S. wittichii* RW1, using specific oligonucleotides (Supplementary Table 5) and introducing suitable restriction sites for cloning. After restriction with the respective enzymes, PCR-amplified DNA fragments were cloned into a pET9a, pET16b, pET17b, pET28a, pBAD24, or a pCDFDuet-1 vector as detailed in Supplementary Table 6.

SPT-encoding plasmids pJPG05, pJPG06 and pJPG07 (Supplementary Table 6) were each digested with *Nde*I and *Bam*HI, and the *spt*-encoding fragments were recloned into pET9a, which had been digested with *Nde*I and *Bam*HI, to yield the pET9a derivatives pJPG08, pJPG01 and pJPG02, respectively. Recloning of the CC_1165 (*aasR*)-encoding *Nde*I/*Bam*HI fragment from pDG04 (Supplementary Table 6) into a *Nde*I/*Bam*HI-restricted pET16b vector yielded plasmid pDG10. The gene encoding acyl-ACP synthetase (*aasS*) from *V. harveyi* (LA59_RS23465) was synthesized by DNA2.0 from ATUM^2^ and subsequently recloned as *Nde*I/*Bam*HI fragment into *Nde*I/*Bam*HI-digested pET16b, to yield plasmid pJPG10. Recloning of the CC_1165 (*aasR*)-encoding *Nco*I/*Bam*HI fragment from pDG10 (Table 1) into a *Nco*I/*Bam*HI-restricted pCDFDuet-1 (MCS-1) vector yielded plasmid pJPG13. Recloning of the CC_1163 (*acpR*)-encoding *Nde*I/*Bam*HI fragment from pDG01 into a *Nde*I/*Bgl*II-restricted pJPG13 (MCS-2) vector yielded plasmid pJPG14. Recloning of the CC_1163/CC_1162 (*acpR/spt*)-encoding *Nde*I/*Bam*HI fragment from pRJ03 (Olea-Ozuna et al., 2021) into a *Nde*I/*Bgl*II-restricted pCDFDuet-1 (MCS-2) vector yielded plasmid pJPG16. Recloning of the *smc02162* (*fadD*)-encoding *Nde*I/*Hind*III fragment from pECH6 (Pech-Canul et al., 2020) into a *Nde*I/*Hind*III-restricted pET28a vector yielded plasmid pECH3.

### Polyacrylamide gel electrophoresis systems for protein analyses

Proteins were analyzed employing different PAGE systems (native PAGE, SDS-PAGE, urea-PAGE, or SDS-Tricine-PAGE) according to necessity. *In vitro* conversions from apo- to holo-ACPs, using the holo-ACP synthase (AcpS) from *S. meliloti* 1021 were analyzed by 20% native PAGE (Jackowski and Rock, 1983). *In vivo* β-[^3^H]alanine incorporation into the 4'-PPT prosthetic group of ACPs was analyzed by 20% native PAGE as well as by SDS-Tricine-PAGE (Schägger and von Jagow, 1987) containing 12% polyacrylamide. This latter system is frequently used for separating peptides and small proteins such as ACPs. Conformation-sensitive urea-PAGE (Post-Beittenmiller et al., 1991), containing 18.7% polyacrylamide, was employed to analyze the *in vitro* acylation of ACPs with fatty acids of different length, using AasR (CC_1165) from *C. crescentus*, and Aas from *V. harveyi*. Different concentrations of urea (up to 5 M) in both the separation and stacking gel were used to separate acyl-ACPs. Furthermore, overexpression and purification of His-tagged proteins was analyzed by denaturing SDS-PAGE (Schägger et al., 1988) containing 12% polyacrylamide.

### *In vivo* labeling of ACPs with β-[^3^H]alanine

*In vivo* labeling of ACPs with β-[^3^H]alanine, the biosynthetic precursor of 4'-phosphopantetheine, was carried out essentially as described previously using the β-alanine auxotrophic strain OG7001 (Epple et al., 1998; López-Lara and Geiger, 2000).

### Expression and purification of presumptive acyl carrier proteins

Strains of *E. coli* BL21(DE3) × pLysS harboring plasmid pDG01 (with specialized *acpR* CC_1163), pPEG01 (with housekeeping *acpP* CC_1677), pJPG12 (with specialized *acpR* ECD_02853), or pTB5079 (with housekeeping *acpP* ECD_01090 of *E. coli*) were grown at 30°C in 1 L of LB medium and at an optical density at 600 nm (OD_600_) of 0.4, isopropyl-β-D-thiogalactoside (IPTG) was added to a final concentration of 0.1 mM to induce protein expression. After induction for 4 h, cells were collected by centrifugation at 7,500 × *g* for 30 min at 4°C, cell pellets were resuspended in 50 ml of buffer A (50 mM Tris/HCl, 0.1 M KCl, pH 6.8) and stored at −20°C. After thawing, the cells were broken by passing cell suspensions twice through a French pressure cell at 20,000 pounds per square inch (psi), the remaining unbroken cells and debris were removed by centrifugation at 7,000 × *g* for 15 min at 4°C and supernatants were used as cell-free extracts (CE).

For the purification of all ACPs, the respective CE was slowly stirred at 4°C and cold 2-propanol was added dropwise to a given CE up to a final concentration of 50% (v/v). After incubation for 60 min at 4°C, the precipitate was removed by centrifugation at 7,000 × *g* for 15 min at 4°C. The 2-propanol-containing supernatant was dialyzed overnight against 2 liter of buffer A. The dialysate was subjected to ion-exchange chromatography using DEAE-52 cellulose (Whatman). Each of the samples was applied to a 30 ml column, that had been equilibrated with two column volumes of buffer B (10 mM Bis-Tris/HCl, pH 6.0, 1 mM 3-cholamidopropyl dimethylammonio 1-propanesulfonate). Each column was washed with two column volumes of buffer B, and proteins were eluted with a linear gradient of NaCl (0.1–1 M) in buffer B in a total volume of 150 ml. Fractions (3 ml) were collected, and aliquots were analyzed in 20% native PAGE (Supplementary Figure 2). ACPs AcpP*_*Cc*_* (CC_1677), AcpP*_*Ec*_* (ECD_01090), and AcpR*_*Ec*_* (ECD_02853) eluted in a range from 0.28 to 0.35 M NaCl, while the ACP AcpR*_*Cc*_* (CC_1163) eluted in a range from 0.21 to 0.28 M NaCl. From each chromatographic separation, five ACP-containing fractions were combined and concentrated using Amicon Ultra-15 Centrifugal Filter Devices (Merck Millipore). Washing steps with 100 mM Tris/HCl, pH 8.0 in the centrifugal device ensured that the final concentration of NaCl was less than 20 mM. The respective ACPs were stored in a final volume of 1.5 ml at −20°C.

### *In vitro* conversion of apo-ACPs to holo-ACPs

To convert purified apo-ACPs to functional holo-ACPs, AcpS*_*Sm*_* obtained from *E. coli* containing plasmid pSBA01 was used as a tool for in *in vitro* reactions employing CoA as phosphopantetheine donor as previously described (Ramos-Vega et al., 2009).

### Expression and purification of acyl-CoA and acyl-ACP synthetases

Strains of *E. coli* BL21(DE3) × pLysS harboring plasmid pDG10 (with specialized *aasR* CC_1165 of *Caulobacter*), pJPG10 (with *aas* of *V. harveyi*), or pECH3 (with *fadD* of *Sinorhizobium*) were grown at 30°C in 25 ml of LB medium and at OD_600_ of 0.4, IPTG was added to a final concentration of 0.1 mM. After induction for 4 h, cells were collected by centrifugation at 7,500 × *g* for 30 min at 4°C and cell pellets were resuspended in 2 ml of binding buffer (20 mM sodium phosphate, 0.5 M NaCl, 20 mM imidazole, pH 7.4). After passing cell suspensions twice through a French pressure cell at 20,000 psi, the remaining unbroken cells and debris were removed by centrifugation at 7,000 × *g* for 15 min at 4°C and supernatants were used as CEs.

The His-tagged proteins were purified using the HiTrap IMAC HP systems (GE Life Sciences) with column bed volumes of 1 ml, respectively. CEs were diluted 1:2 with binding buffer and applied to a chromatography column previously equilibrated with 10 ml of binding buffer. Then columns were washed with 10 ml of binding buffer. Stepwise elution was performed with portions of 5 ml of binding buffer containing increased concentrations of imidazole (100, 250, 500, 750 mM). Fractions were collected and analyzed in 12% SDS-PAGE (Supplementary Figure 4). Fractions containing the respective His-tagged protein (His_10_-tagged putative AasR CC_1165, His_10_-tagged Aas from *V. harveyi* (Aas*_*Vh*_*) and His_6_-tagged FadD from *S. meliloti*) were near homogeneous and were dialyzed against Aas buffer (20 mM Tris-HCl, pH 7.5, 10% glycerol, 1 mM EDTA, 0.1 mM DTT, and 0.002% Triton X-100), similarly, as described by Jiang et al. (2006). The protein content of homogeneous acyl-CoA and acyl-ACP synthetase preparations was determined using the method reported by Dulley and Grieve (1975).

### Enzymatic assays for acyl-CoA and acyl-ACP synthetases

The enzymatic assays for acyl-CoA and acyl-ACP synthetases described here are essentially modified versions of the assay reported by Jiang et al. (2006) for acylating ACPs with acyl-ACP synthetase from *V. harveyi*.

For each reaction, 10 nmol [9,10-^3^H]-palmitic acid (specific radioactivity: 743 mCi/mmol) in ethanolic solution and an equimolar amount of NaOH were combined with an aqueous solution containing 35 μg Triton X-100 in an Eppendorf tube and solvents were removed under vacuum. Aqueous Tris/HCl buffer, pH 8.0 was added, the tubes were vortexed three times for 30 s and sonicated for 30 min. In a total volume of 50 μl, the final assay contained 200 μM Na [^3^H]-palmitate (7.4 μCi), 1 mM Triton X-100, 100 mM Tris/HCl buffer, pH 8.0, 10 mM ATP, 10 mM MgSO_4_, 5 mM dithiothreitol, 40 μM thiol substrate (ACPs or CoA) and different concentrations of each enzyme (30 nM for His_10_-AasR*_*Cc*_* (CC_1165), 100 nM for His_10_-Aas*_*Vh*_*, and 300 nM for His_6_-FadD*_*Sm*_*). The reactions were incubated for 0, 15, 30, 45 or 60 min at 37°C in a water bath. Holo-ACPs were quantified by detecting their single sulfhydryl group of 4'-phosphopantetheine using Ellman’s method (Ellman, 1959).

When assaying for acyl-CoA synthetase activities, the reactions were stopped by adding 125 μl of isopropanol/n-heptane/1 M H_2_SO_4_ (40:10:1; v/v). Addition of 25 μl of distilled water and 125 μl n-heptane and thorough mixing led to the separation of the organic and aqueous phases. Subsequently, the organic phase (containing unbound [^3^H]palmitate) was removed and the aqueous phase (containing [^3^H]palmitoyl-CoA products) was washed 6 more times with 125 μl of n-heptane. Radioactivity associated with the aqueous phase ([^3^H]palmitoyl-CoA) was quantified in a scintillation counter.

When assaying for acyl-ACP synthetase activities, the reactions were stopped by freezing them rapidly at −80°C. Subsequently, defrosted samples were applied to Whatman 3MM cellulose filters, washed three times with methanol/chloroform/acetic acid (2:1:0.3; v/v) in a vacuum manifold. Filters were dried and filter-bound radioactivity was quantified in a scintillation counter, similarly, as described by Ray and Cronan (1976).

### Kinetic analysis of acyl-AcpR synthetase AasR

In order to study the effect of the AcpR substrate concentration on AasR activity, initial velocities for holo-AcpR*_*Cc*_* (CC_1163) acylation were determined at different AcpR*_*Cc*_* concentrations (2.5, 5, 10, 20, 30, 40, 50, 60, 70, 80, 90, and 100 μM) employing His_10_-AasR*_*Cc*_* (CC_1165) and [^3^H]palmitate. Samples were incubated at 37°C in a water bath for 30 min, period for which product formation was linear for all AcpR concentrations employed. Subsequently, the initial velocities (V_0_) were calculated measuring the slope (m) of the incorporation of [^3^H]palmitate into the product [^3^H]palmitoyl-AcpR*_*Cc*_* and plotted against the concentration of the holo-AcpR (CC_1163) substrate (Figure 6).

### Expression of serine palmitoyltransferases

Strains of *E. coli* BL21(DE3) × pLysS harboring plasmid pJPG08 (with *spt* CC_1162 of *C. crescentus*), pJPG01 (with *spt* ECD_02854 of *E. coli* BL21(DE3)), pJPG02 (with *spt* Swit_3900 of *S. wittichii*), or the empty pET9a vector were grown at 30°C in 25 ml of LB medium and at OD_600_ of 0.4, IPTG was added to a final concentration of 0.1 mM. After induction for 4 h, cells were collected by centrifugation at 7,500 × *g* for 30 min at 4°C and cell pellets were resuspended in 2 ml of buffer containing 20 mM potassium phosphate (pH 7.4), 150 mM NaCl, 5 mM DTT (Raman et al., 2009). After passing cell suspensions twice through a French pressure cell at 20,000 psi, the remaining unbroken cells and debris were removed by centrifugation at 7,000 × *g* for 15 min at 4°C and supernatants were used as CEs.

### Serine palmitoyltransferases enzymatic assays

Enzymatic assays for serine palmitoyltransferase were performed similarly, to those described by Ikushiro et al. (2001). Each assay contained in a final volume of 100 μl, 100 mM HEPES/NaOH (pH 7.5), 100 μM EDTA, 5 mM DTT, 10 μM pyridoxal phosphate, and 432 μM (0.2 μCi) of L-[^14^C(U)]serine (PerkinElmer; specific radioactivity: 46.4 mCi/mmol). Thioester substrates (palmitoyl-CoA, palmitoyl-AcpR*_*Cc*_* or palmitoyl-AcpP*_*Cc*_* were added to a final concentration of 10 μM. For each 100 μl assay, 10 μg protein of CEs from *E. coli* BL21(DE3) × pLysS carrying the empty pET9a vector, or SPT-expressing plasmids were employed respectively. Enzyme assays were incubated at 30°C for 4 h in water bath and the reactions were stopped by adding 100 μl of NH_4_OH. For each sample, separation of the organic and aqueous phases was carried out by adding 375 μl of chloroform/methanol (2:1; v/v) (Bligh and Dyer, 1959). After centrifugation at 16,000 × *g* for 5 min, the organic phases were transferred to fresh tubes and dried (Raman et al., 2009). The dried lipids were dissolved in 20 μl of chloroform/methanol (2:1; v/v) and spotted onto a thin layer chromatography (TLC) plate (Silica Gel 60, Merck). Separation in one dimension (1D) was carried out with a mobile phase of chloroform/methanol/2N ammonium hydroxide (40:10:1; v/v). The developed chromatogram was exposed to Phosphor Screens (GE Healthcare), and the autoradiograms were visualized with an optical scanner (Typhoon FLA 9500 from GE Healthcare Life Sciences).

### Thin-layer chromatographic analyses of radioactive samples and sphingolipid intermediate standards

For all figures obtained using TLC (Figures 7, 8 and Supplementary Figure 8), relative mobilities of non-radioactive standard samples and radioactive experimental samples were compared after they had been separated on the same TLC plate. However, as the radioactive and non-radioactive samples needed to be detected by different methods, the aluminum-backed, developed silica plates were cut with scissors in order to visualize radioactive samples by autoradiography and non-radioactive samples by iodine staining, as indicated in the corresponding figure legends. Based on the reconstituted silica plates, the two image subparts can be clearly assembled. The subparts of the recomposed figures are surrounded by black squares.

### Mass spectrometric analyses of acyl carrier proteins

Acylation reactions for mass spectrometric analyses were as previously described in “Enzymatic assays for acyl-CoA and acyl-ACP synthetases.” However, the acylations were performed in the absence of Triton X-100 and with 300 nM of each enzyme (AasR*_*Cc*_*, or Aas*_*Vh*_*).

ACP preparations were precipitated with acetone at −20°C and washed twice with cold acetone. After drying, ACP-containing samples were dissolved in water and of each sample 2.5 μg of protein were injected into a LCQ fleet ion trap mass spectrometer (Thermo Fisher Scientific, Waltham, MA, United States). Spray voltage was set to 5 kV, sheath gas flow rate at 20 units and the capillary temperature at 250°C.

Before injection into the mass spectrometer, acyl- (palmitoyl-) AcpP*_*Cc*_* was subjected to chromatographic separation in an Ultimex 3000 HPLC system (Dionex, Sunnyvale, CA, United States) equipped with a Hypersil Gold C18 (100 × 2.1 mm) analytical column (Thermo Fisher Scientific).

Spectra visualization was performed using Xcalibur software (Thermo Fisher Scientific) and MZmine 2.38 (Pluskal et al., 2010). Multiple charge states were observed for each of the major species of each protein. The mass of each protein was calculated using ESIprot (Winkler, 2010).

## Data availability statement

The original contributions presented in the study are included in the article/Supplementary material, further inquiries can be directed to the corresponding author/s.

## Author contributions

JP-G, DS-C, SP, SE-G, IL-L, and OG designed the study. JP-G, RO-O, DG-S, SC-M, and IL-L carried out the experiments. JP-G, RO-O, SP, OM-T, IL-L, and OG carried out the data analysis and discussed the results. JP-G, SE-G, IL-L, and OG were involved in drafting the manuscript and all authors read and approved the final manuscript.

## Footnotes

1
https://www.expasy.org/

2
www.atum.bio
