# Supplementary material for: Specialized acyl carrier protein used by serine palmitoyltransferase to synthesize sphingolipids in Rhodobacteria
Source: Front Microbiol. 2022 Aug 4;13:961041. doi: 10.3389/fmicb.2022.961041 (PMC9386255; doi:10.3389/fmicb.2022.961041)
Supplement: Supplementary file 1 [file Data_Sheet_1.pdf]

Specialized acyl carrier protein used by serine palmitoyltransferase to synthesize sphingolipids in *Rhodobacteria*

Supplementary Figures

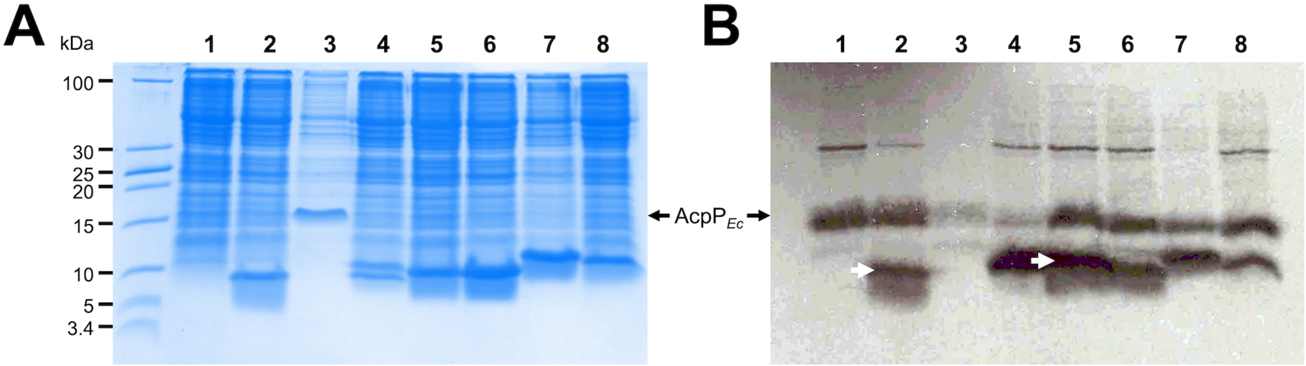

Figure S1. ***In vivo*** **labeling of ACPs with β-[^{3}H]alanine.** Proteins of cell-free extracts from *E. coli* OG7001 x pLysS strains overproducing different ACPs from pET9a-derived plasmids were separated in a 12% SDS-Tricine PAGE. (A) Proteins stained with Coomassie blue and (B) autoradiogram visualized after labeling with β-[^{3}H]alanine. In (A) and (B), extracts of *E. coli* carrying the pET9a vector (lane 1), $AcpR_{Ec}$ (ECD_02853)-expressing pJPG12 (lane 2), $AcpP_{Ec}$ (ECD_01090)-expressing pTB5079 (lane 3), $AcpP_{Cc}$ (CC_1677)-expressing pPEG01 (lane 4), $AcpR_{Cc}$ (CC_1163)-expressing pDG01 (lane 5), NodF-expressing pMP2301 (lane 6), AcpXL-expressing pAL07 (lane 7) or RkpF-expressing pTB1003 (lane 8) were analyzed. White arrows (B) highlight radiolabeled specialized ACPs $AcpR_{Ec}$ and $AcpR_{Cc}$ from *E. coli* and *C. crescentus*, respectively. PageRuler Low Range Unstained Protein Ladder (Thermo Scientific) is displayed (A).

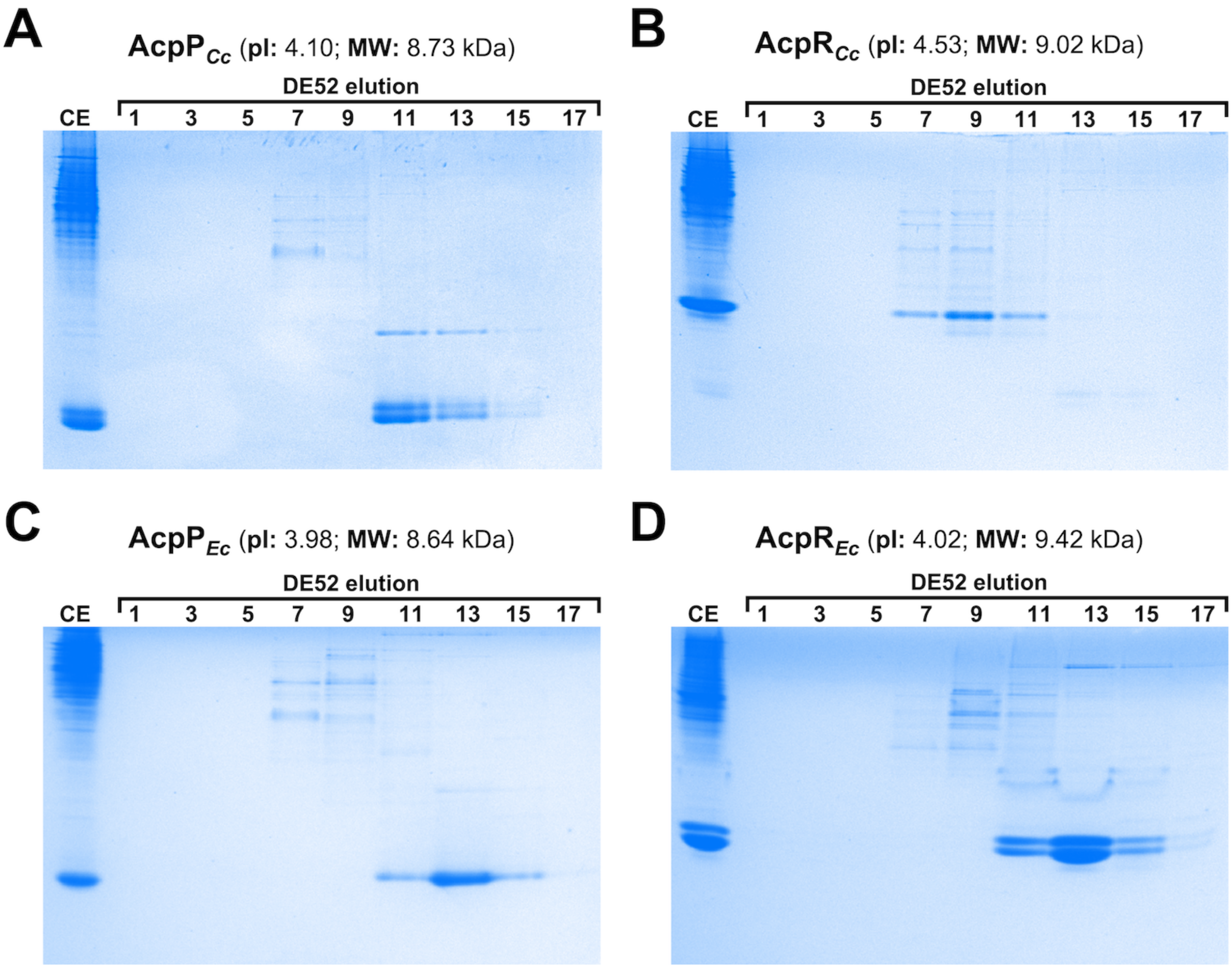

Figure S2. Purification of ACPs AcpP$_{Cc}$, AcpR$_{Cc}$, AcpP$_{Ec}$ and AcpR$_{Ec}$ by ion-exchange chromatography. Cell-free extracts (CE), containing overexpressed ACPs (AcpP$_{Cc}$ from pPEG01 (A), AcpR$_{Cc}$ from pDG01 (B), AcpP$_{Ec}$ from pTB5079 (C) and AcpR$_{Ec}$ from pJPG12 (D)), and selected fractions eluted during chromatographies on DE52-cellulose columns (fraction number 1, 3, 5, 7, 9, 11, 13, 15, and 17) were separated in 20% native PAGE and stained with Coomassie blue. Isoelectric point (pI) and molecular weight (MW) are indicated for each ACP and were calculated using the ExPASy - SIB Bioinformatics Resource Portal (https://www.expasy.org/).

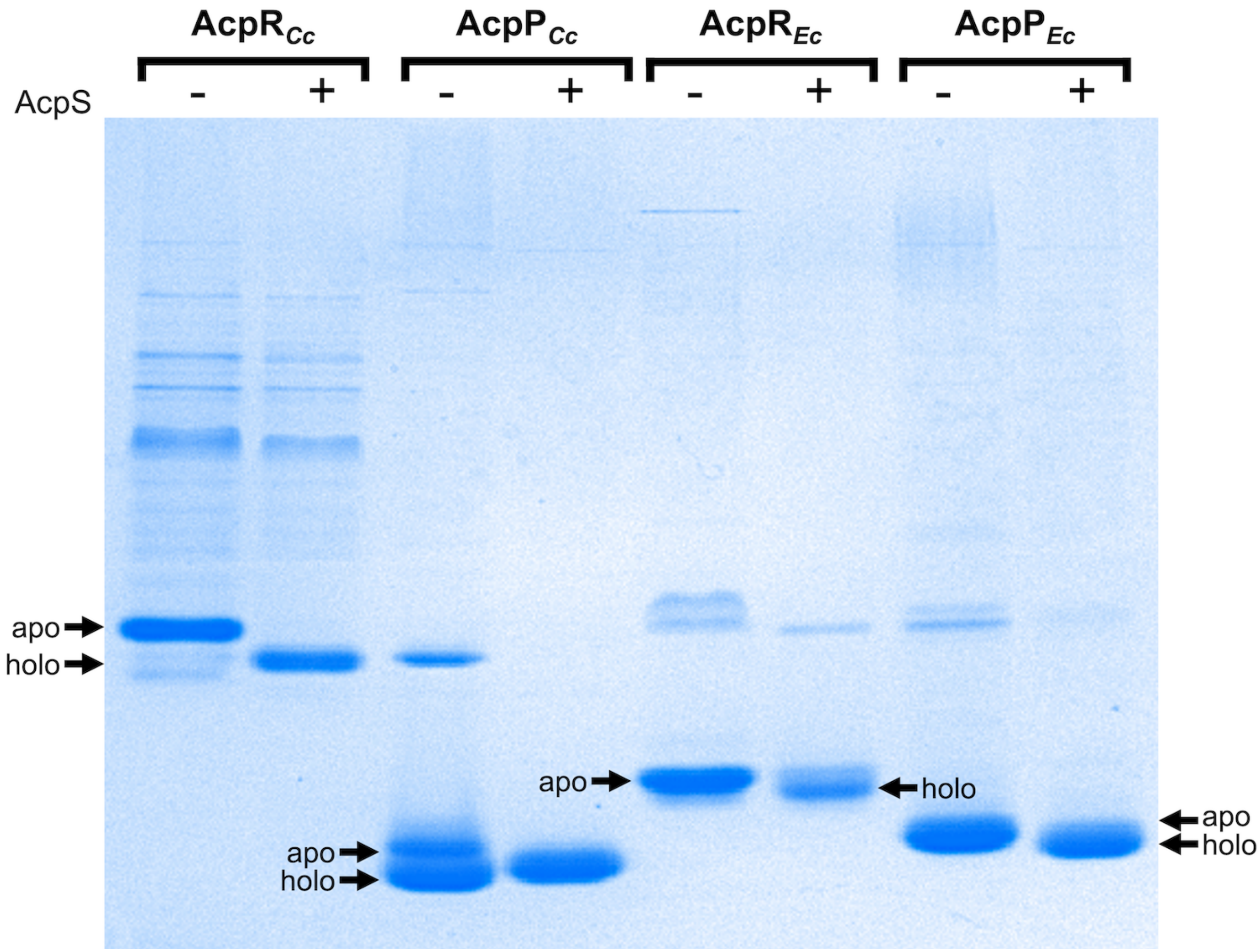

Figure S3. *In vitro* conversion of ACPs from apo- to holo-forms. Purified ACP preparations containing mixtures of apo- and holo-ACPs (AcpR$_{Cc}$ and AcpP$_{Cc}$ from *C. crescentus*; and AcpR$_{Ec}$ and AcpP$_{Ec}$ from *E. coli* BL21(DE3)) were incubated with CoA in the presence of holo-ACP synthase (AcpS) from *S. meliloti*. A Coomassie-blue stained gel of native 20% PAGE containing purified ACP preparations without (-) or with (+) AcpS incubation is displayed. The positions of apo- and holo-forms for each ACP are indicated.

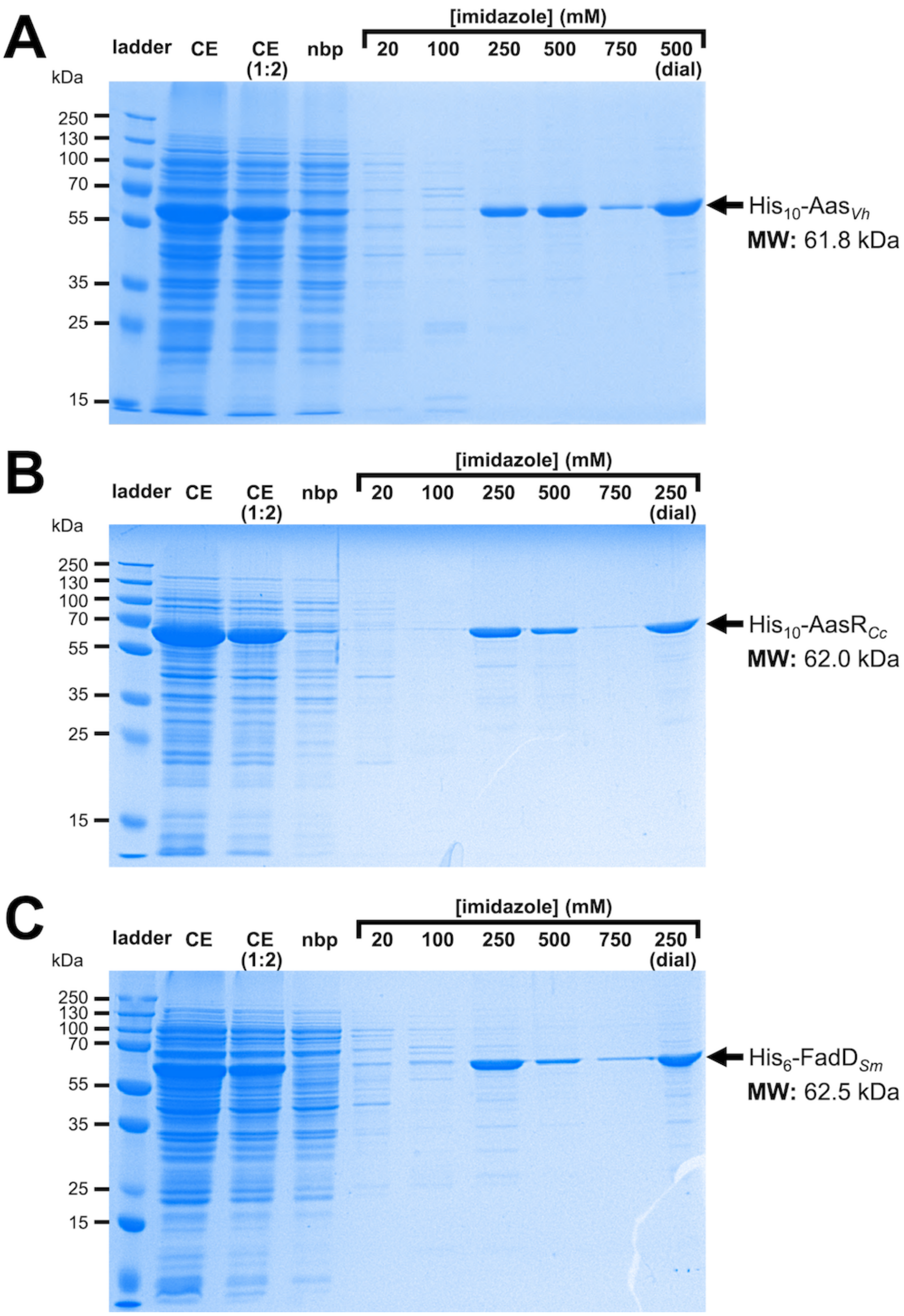

Figure S4. Purification of His-tagged acyl-CoA/ACP synthetases by affinity chromatography. Cell-free extracts, containing overexpressed His-tagged acyl-CoA/ACP synthetases, and fractions obtained by Ni-containing HiTrap column chromatography were separated in 12% SDS-PAGE and stained with Coomassie blue. Fractions of chromatographic elutions of the Aas_{Vh} from *V. harveyi* (A), putative ACS $AasR_{Cc}$ from *C. crescentus* (B) and $FadD_{Sm}$ from *S. meliloti* (C) are shown. The samples included PageRuler Plus Prestained Protein ladder from Thermo Scientific (ladder), cell-free extract (CE), diluted CE (1:2), proteins not bound to HiTrap column (nbp), and elution fractions obtained with different concentrations (20, 100, 250, 500 and 750 mM) of imidazole. An eluted fraction after dialysis (dial) is shown for each protein. The molecular weight (MW) for each protein was calculated using the ExPASy - SIB Bioinformatics Resource Portal (https://www.expasy.org/).

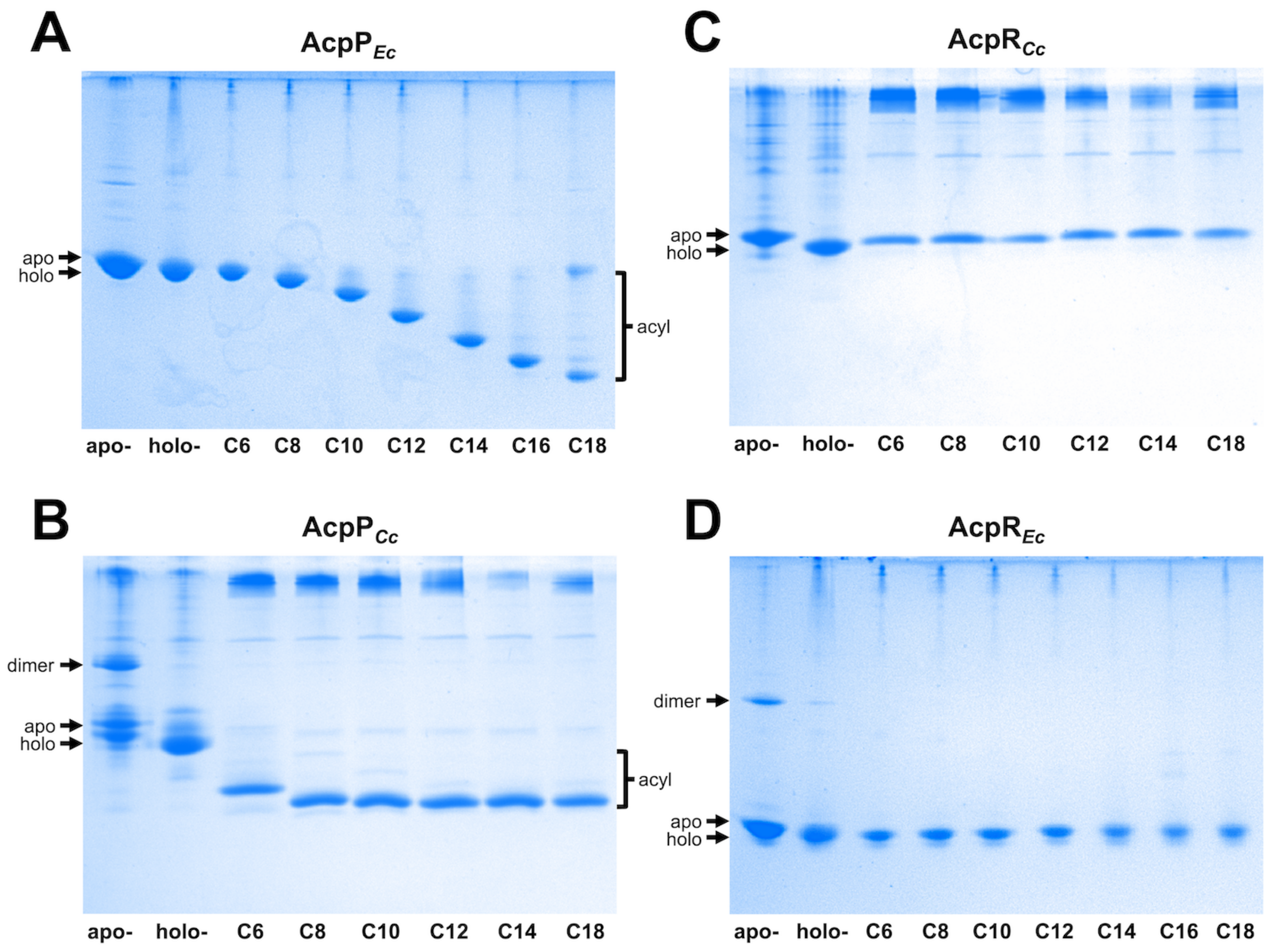

Figure S5. Acylation assays (with C6-C18 fatty acids) of different ACPs, using Aas from *V. harveyi*. ACP preparations of AcpP$_{Ec}$ (A), AcpP$_{Cc}$ (B), AcpR$_{Cc}$ (C) and AcpR$_{Ec}$ (D). Preparations after ion exchange chromatography (apo-ACP; apo-), after treatment with AcpS (holo-ACP; holo-), or after subsequent acylation treatment with Aas$_{Vh}$ and selected fatty acids (hexanoic acid (C6), octanoic acid (C8), decanoic acid (C10), lauric acid (C12), myristic acid (C14), palmitic acid (C16), and stearic acid (C18) were separated by conformation-sensitive urea-PAGE and stained with Coomassie blue. Samples in (A) and (D) were separated in 17.3% (5 M urea) PAGE, and samples in (B) and (C) in 18.7% (3.2 M urea) PAGE. Reactions were performed at 37°C in a water bath during 18 h. Different isoforms of the ACPs are shown: apo-, holo-, acyl-ACP, and dimers.

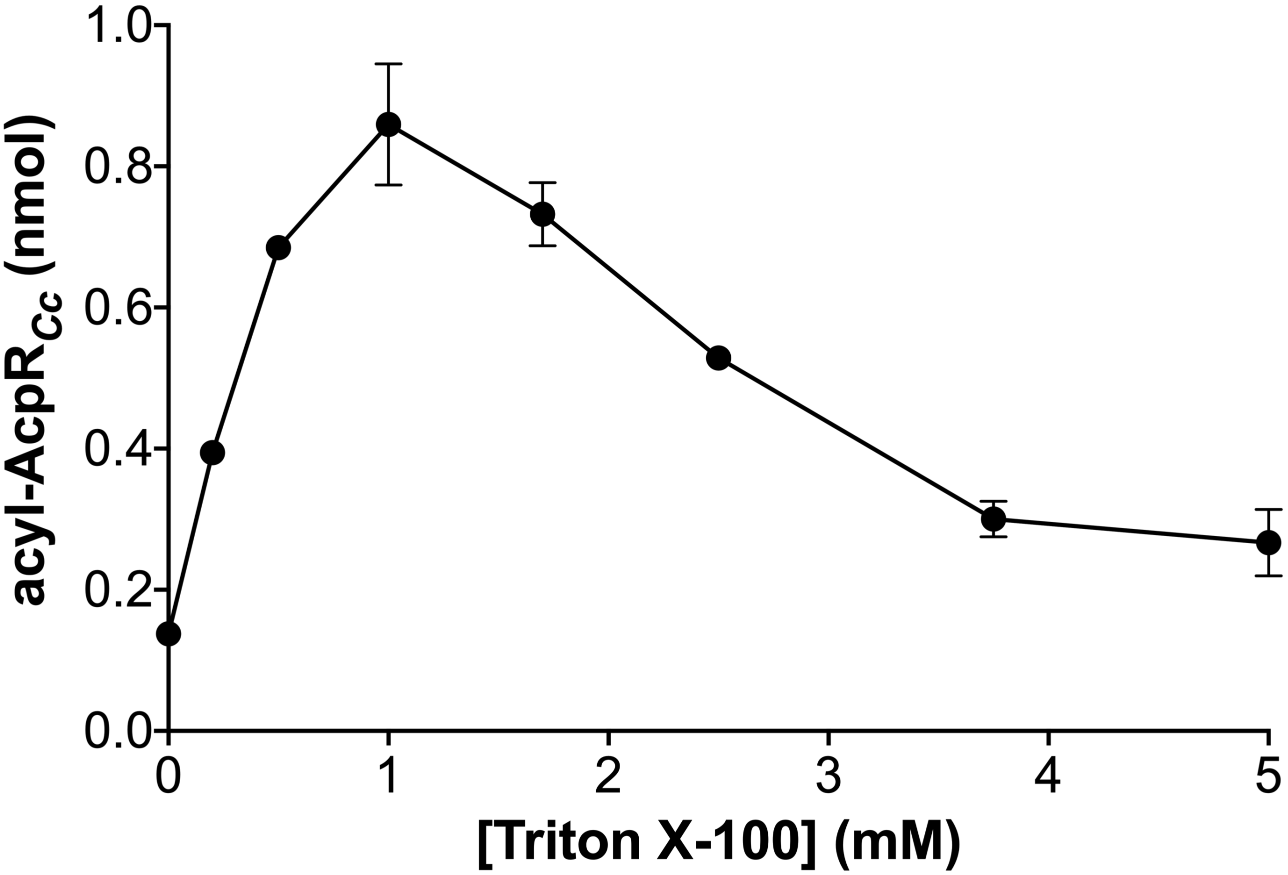

Figure S6. Dependence of AasR activity on Triton X-100 concentration. Holo-AcpR$_{Cc}$ acylation with AasR$_{Cc}$ from *C. crescentus* was assayed using [^{3}H]palmitate and different concentrations (0, 0.2, 0.5, 1, 1.7, 2.5, 3.75, or 5 mM) of the nonionic detergent Triton X-100. Activity was measured by analyzing the incorporation of [^{3}H]palmitate into the [^{3}H]palmitoyl-AcpR$_{Cc}$ product, as described in Materials and methods. Assays were performed at 37ºC in a water bath during 30 minutes. Concentrations of 40 µM thiol substrate holo-AcpR$_{Cc}$ and 30 nM of AasR$_{Cc}$ were employed. Standard deviations of three replicates are shown.

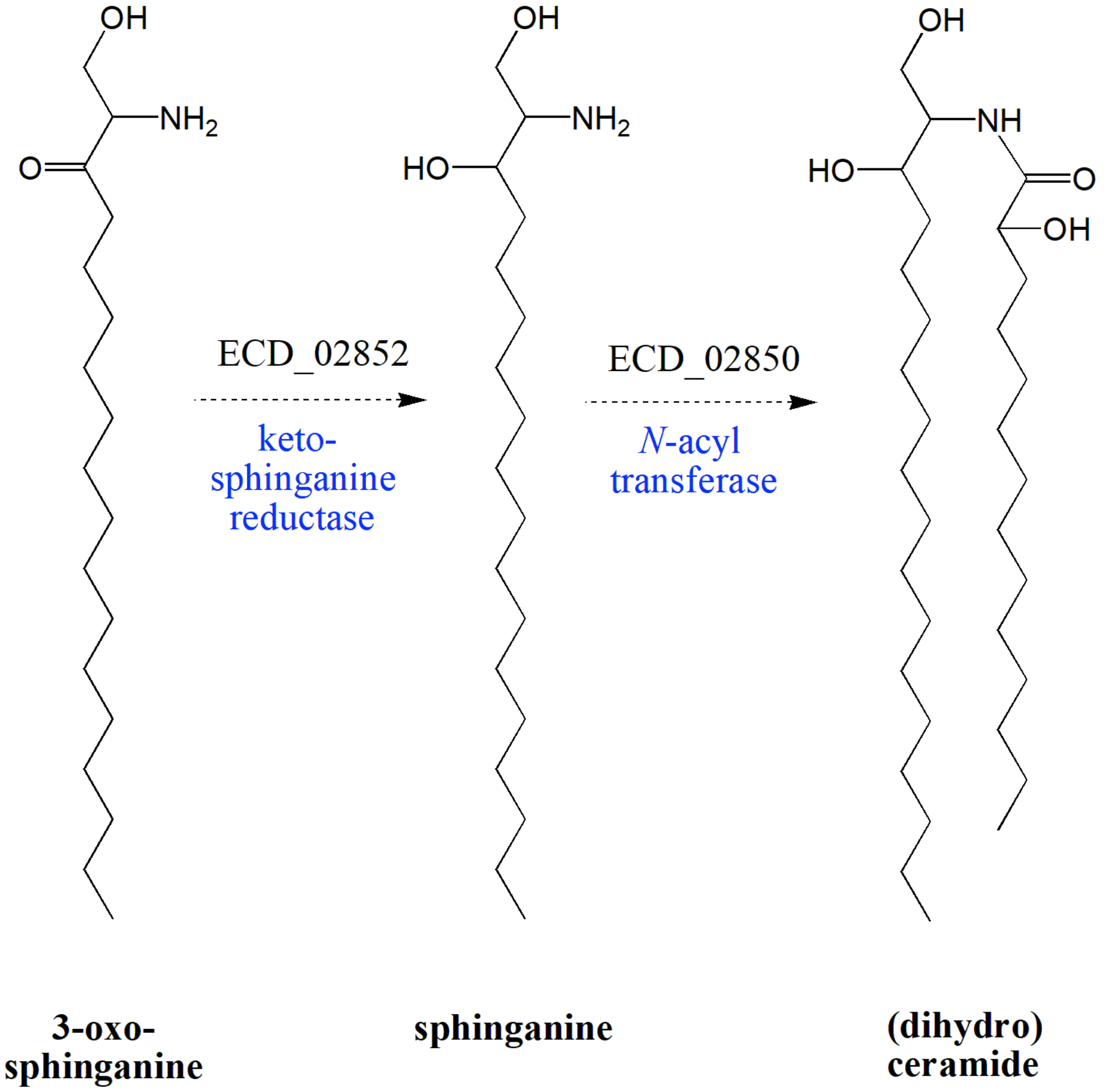

Figure S7. Proposed 3-oxo-sphinganine conversion in *E. coli*. 3-oxo-sphinganine formed in *E. coli* BL21(DE3) might be converted by intrinsic enzymes to sphinganine and (dihydro)ceramide.

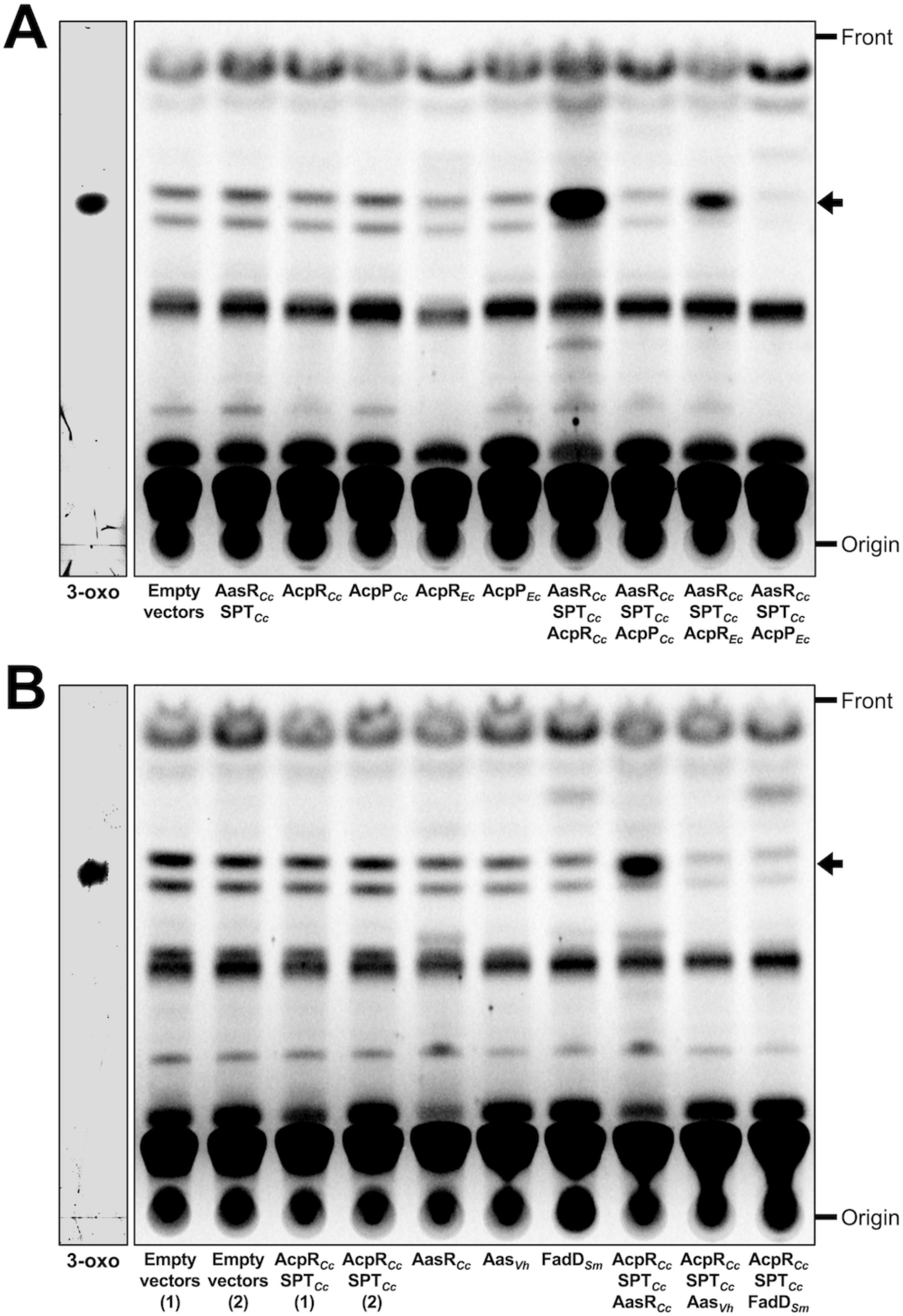

Figure S8. Specialized acyl-ACP synthetase AasR, specialized AcpR, and serine palmitoyltransferase from *C. crescentus* are required for efficient 3-oxo-sphinganine formation. Expression of the structural genes for SPT_{Cc} and $AasR_{Cc}$ from *C. crescentus* in combination with any of the structural genes for the constitutive or specialized ACP from *C. crescentus* ($AcpP_{Cc}$ $AcpR_{Cc}$) or *E. coli* BL21(DE3) ($AcpP_{Ec}$ $AcpR_{Ec}$) leads to the strong formation of a compound that migrates like 3-oxo-sphinganine in TLC when specialized $AcpR_{Cc}$ is employed and to reduced formation if $AcpR_{Ec}$ is used instead. Radiolabeling with ^{14}C-acetate was performed on complex medium at 30ºC for 4 h (transition of exponential to stationary phase of growth) after induction with IPTG at an

OD_{600} = 0.3 with *E. coli* BL21(DE3) x pLysS expressing different sphingolipid biosynthesis genes, as described previously (Olea-Ozuna et al., 2021). Strains of *E. coli* BL21(DE3) x pLysS employed harbored (A) the empty vectors pCDFDuet-1 and pET9a (Empty vectors), pJPG15 and pET9a ($AasR_{Cc}$ SPT_{Cc}), pCDFDuet-1 and pDG01 ($AcpR_{Cc}$), pCDFDuet-1 and pPEG01 ($AcpP_{Cc}$), pCDFDuet-1 and pJPG12 ($AcpR_{Ec}$), pCDFDuet-1 and pTB5079 ($AcpP_{Ec}$), pJPG15 and pDG01 ($AasR_{Cc}$ SPT_{Cc} $AcpR_{Cc}$), pJPG15 and pPEG01 ($AasR_{Cc}$ SPT_{Cc} $AcpP_{Cc}$), pJPG15 and pJPG12 ($AasR_{Cc}$ SPT_{Cc} $AcpR_{Ec}$), and pJPG15 and pTB5079 ($AasR_{Cc}$ SPT_{Cc} $AcpP_{Ec}$). (B) Strains of *E. coli* BL21(DE3) x pLysS employed harbored the empty vectors pCDFDuet-1 and pET16b (Empty vectors (1)), pCDFDuet-1 and pET28a (Empty vectors (2)), pJPG16 and pET16b ($AcpR_{Cc}$ SPT_{Cc} (1)), pJPG16 and pET28a ($AcpR_{Cc}$ SPT_{Cc} (2)), pCDFDuet-1 and pDG10 ($AasR_{Cc}$), pCDFDuet-1 and pJPG10 (Aas_{Vh}), pCDFDuet-1 and pECH3 ($FadD_{Sm}$), pJPG16 and pDG10 ($AcpR_{Cc}$ SPT_{Cc} $AasR_{Cc}$), pJPG16 and pJPG10 ($AcpR_{Cc}$ SPT_{Cc} Aas_{Vh}), and pJPG16 and pECH3 ($AcpR_{Cc}$ SPT_{Cc} $FadD_{Sm}$). At the end of the labeling period, cells were harvested, lipids were extracted, separated by TLC and developed chromatograms were subjected to autoradiography. The arrow indicates a compound migrating like 3-oxo-sphinganine. The reference compound 3-oxo-sphinganine (3-oxo), was developed in the same TLC and visualized by iodine staining.

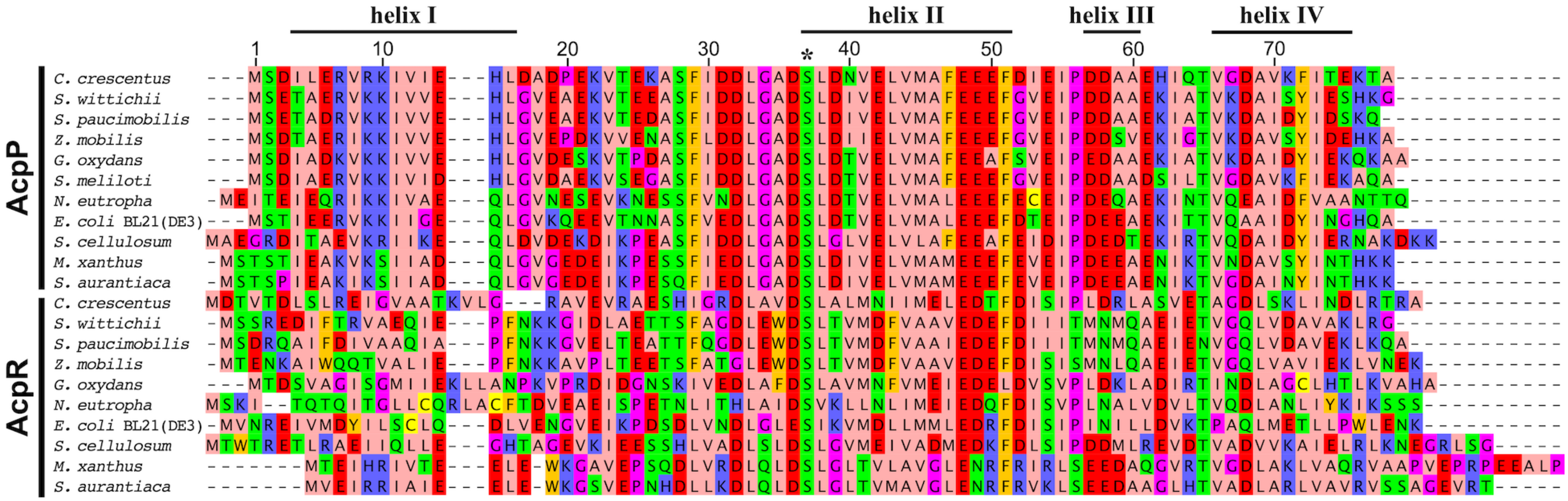

Figure S9. Multiple sequence alignments between housekeeping AcpPs and specialized AcpRs. Amino acid sequences of distinct ACPs (acyl carrier proteins) from Rhodobacteria and δ-Proteobacteria were aligned using the CLUSTAL OMEGA program (https://www.ebi.ac.uk/Tools/msa/clustalo/) and shaded using Jalview (Waterhouse et al., 2009). Conserved residues are shown in equal colors. The 4 α-helices and position numbers corresponding to the sequence of *C. crescentus* AcpP are indicated. The asterisk (*) highlightes the attachment site (S37) of the 4'-PPT prosthetic group. ORF names/accession numbers are mentioned in Fig. 10.

References for Supplementary Figures

Olea-Ozuna, R. J., Poggio, S., EdBergström, Quiroz-Rocha, E., García-Soriano, D. A., Sahonero-Canavesi, D. X., Padilla-Gómez, J., Martínez-Aguilar, L., López-Lara, I. M., Thomas-Oates, J., and Geiger, O. (2021). Five structural genes required for ceramide synthesis in *Caulobacter* and for bacterial survival. *Environ Microbiol,* 23**,** 143-159. doi: 10.1111/1462-2920.15280

Waterhouse, A. M., Procter, J. B., Martin, D. M., Clamp, M., & Barton, G. J. (2009). Jalview Version 2--a multiple sequence alignment editor and analysis workbench. *Bioinformatics,* 25**,** 1189-91. doi: 10.1093/bioinformatics/btp033
