# Supplementary material for: Specialized acyl carrier protein used by serine palmitoyltransferase to synthesize sphingolipids in Rhodobacteria
Source: Front Microbiol. 2022 Aug 4;13:961041. doi: 10.3389/fmicb.2022.961041 (PMC9386255; doi:10.3389/fmicb.2022.961041)
Supplement: Supplementary file 2 [file Data_Sheet_2.pdf]

# Specialized acyl carrier protein used by serine palmitoyltransferase to synthesize sphingolipids in *Rhodobacteria*

## Supplementary Tables

**Table S1.** Theoretical and measured masses of acyl carrier proteins AcpR$_{Cc}$ and AcpP$_{Cc}$

| Protein species | **AcpR$_{Cc}$** | | | **AcpP$_{Cc}$** | | |
|---|---|---|---|---|---|---|
| | Detected mass (Da) | Theoret. mass* (Da) | Error (ppm) | Detected mass (Da) | Theoret. mass* (Da) | Error (ppm) |
| **Apo** | 9020.3 | 9022.4 | 232 | 8599.1 | 8601.7 | 302 |
| **Holo** | 9360.8 | 9361.4 | 64 | 8939.8 | 8940.7 | 101 |
| **Palmitoyl** | 9601.2 | 9599.8 | -145 | 9180.3 | 9179.1 | -130 |

*The theoretical masses were determined using https://www.expasy.org and in the case of AcpP$_{Cc}$ without the N-terminal methionine.

**Table S2.** Orthologues for sphingolipid biosynthesis proteins in the *Rhodobacteria C. crescentus*, *E. coli* B21(DE3), *S. wittichii*, *S. paucimobilis*, *Z. mobilis*, *G. oxydans*, and *N. eutropha*.

| **ORF (Query)** | **ORF (Subject)** | **E Value** | **identity (%)** | **similarity (%)** | **coverage (%)** |
|---|---|---|---|---|---|
| $CC\_1154_{Cc}$ (Aca) | $ECD\_02850_{Ec}$ | $1e^{-107}$ | 44 | 61 | 98 |
| $CC\_1154_{Cc}$ (Aca) | $Swit\_3908_{Sw}$ | $1e^{-104}$ | 45 | 58 | 97 |
| $CC\_1154_{Cc}$ (Aca) | $DRN02\_007910_{Sp}$ | $3e^{-91}$ | 41 | 59 | 97 |
| $CC\_1154_{Cc}$ (Aca) | $ZMO1400_{Zm}$ | $7e^{-93}$ | 40 | 57 | 98 |
| $CC\_1154_{Cc}$ (Aca) | $GOX2417_{Go}$ | $9e^{-62}$ | 34 | 50 | 97 |
| $CC\_1154_{Cc}$ (Aca) | $Neut\_1100_{Ne}$ | $2e^{-100}$ | 41 | 58 | 100 |
| $CC\_1162_{Cc}$ (Spt) | $ECD\_02854_{Ec}$ | $4e^{-144}$ | 53 | 68 | 96 |
| $CC\_1162_{Cc}$ (Spt) | $Swit\_3900_{Sw}$ | $1e^{-152}$ | 53 | 73 | 97 |
| $CC\_1162_{Cc}$ (Spt) | $DRN02\_007885_{Sp}$ | $2e^{-147}$ | 53 | 70 | 97 |
| $CC\_1162_{Cc}$ (Spt) | $ZMO1270_{Zm}$ | $3e^{-151}$ | 52 | 72 | 97 |
| $CC\_1162_{Cc}$ (Spt) | $GOX2056_{Go}$ | $1e^{-159}$ | 55 | 72 | 97 |
| $CC\_1162_{Cc}$ (Spt) | $Neut\_0461_{Ne}$ | $1e^{-133}$ | 51 | 66 | 97 |
| $CC\_1163_{Cc}$ (AcpR) | $ECD\_02853_{Ec}$ | $3e^{-08}$ | 41 | 68 | 61 |
| $CC\_1163_{Cc}$ (AcpR) | $Swit\_3899_{Sw}$ | $6e^{-10}$ | 32 | 54 | 71 |
| $CC\_1163_{Cc}$ (AcpR) | $DRN02\_007880_{Sp}$ | $1e^{-09}$ | 32 | 52 | 71 |
| $CC\_1163_{Cc}$ (AcpR) | $ZMO2012_{Zm}$ | $5e^{-12}$ | 38 | 57 | 68 |
| $CC\_1163_{Cc}$ (AcpR) | $GOX2057_{Go}$ | $1e^{-12}$ | 46 | 69 | 67 |
| $CC\_1163_{Cc}$ (AcpR) | $Neut\_0460_{Ne}$ | $8e^{-13}$ | 44 | 71 | 71 |
| $CC\_1164_{Cc}$ (Epi) | $ECD\_02852_{Ec}$ | $7e^{-38}$ | 34 | 49 | 94 |
| $CC\_1164_{Cc}$ (Epi) | $Swit\_3897_{Sw}$ | $2e^{-47}$ | 40 | 47 | 95 |
| $CC\_1164_{Cc}$ (Epi) | $DRN02\_007875_{Sp}$ | $7e^{-37}$ | 35 | 48 | 95 |
| $CC\_1164_{Cc}$ (Epi) | $ZMO0205_{Zm}$ | $1e^{-38}$ | 31 | 48 | 96 |
| $CC\_1164_{Cc}$ (Epi) | $GOX1849_{Go}$ | $2e^{-18}$ | 32 | 47 | 73 |
| $CC\_1164_{Cc}$ (Epi) | $Neut\_0459_{Ne}$ | $1e^{-47}$ | 38 | 52 | 85 |
| $CC\_1165_{Cc}$ (AasR) | $ECD\_02851_{Ec}$ | $1e^{-155}$ | 42 | 60 | 97 |
| $CC\_1165_{Cc}$ (AasR) | $Swit\_2559_{Sw}$ | $1e^{-163}$ | 46 | 63 | 98 |
| $CC\_1165_{Cc}$ (AasR) | $DRN02\_008180_{Sp}$ | $7e^{-163}$ | 46 | 62 | 98 |
| $CC\_1165_{Cc}$ (AasR) | $ZMO0704_{Zm}$ | $3e^{-164}$ | 45 | 63 | 98 |
| $CC\_1165_{Cc}$ (AasR) | $GOX2058_{Go}$ | 0 | 54 | 69 | 99 |
| $CC\_1165_{Cc}$ (AasR) | $Neut\_0458_{Ne}$ | $6e^{-152}$ | 43 | 60 | 99 |

Pairwise protein sequence alignments between *C. crescentus* ($_{Cc}$) ORFs codifying proteins for dihydroceramide biosynthesis: putative acyl-CoA *N*-acyltransferase (Aca), serine palmitoyltransferase (Spt), acyl carrier protein (AcpR), predicted dehydrogenase/epimerase (Epi), and acyl-ACP synthetase (AasR); compared to the *E. coli* BL21(DE3) ($_{Ec}$), *S. wittichii* ($_{Sw}$), *S. paucimobilis* ($_{Sp}$), *Z. mobilis* ($_{Zm}$), *G. oxydans* ($_{Go}$), and *N. eutropha* ($_{Ne}$) homologues. Homologous ORFs are shown in equal colors. ORF names/accession numbers are as follows: *Caulobacter crescentus* CB15 (Aca: CC_1154/AAK23138.1; Spt: CC_1162/AAK23146.1; AcpR: CC_1163/AAK23147.1; Epi: CC_1164/AAK23148.1; AasR: CC_1165/AAK23149.1), *Escherichia coli* BL21(DE3) (Aca: ECD_02850/ACT44654.1; Spt: ECD_02854/ACT44658.1; AcpR: ECD_02853/ACT44657.1; Epi: ECD_02852/ACT44656.1; AasR: ECD_02851/ACT44655.1), *Sphingomonas wittichii* RW1 (Aca: Swit_3908/ABQ70253.1; Spt: Swit_3900/ABQ70245.1; AcpR: Swit_3899/ABQ70244.1; Epi: Swit_3897/ABQ70242.1; AasR: Swit_2559/ABQ68917.1), *Sphingomonas paucimobilis* strain AIMST S-2 (Aca: DRN02_007910/QBE91945.1; Spt:

DRN02_007885/QBE91942.1; AcpR: DRN02_007880/QBE91941.1; Epi: DRN02_007875/QBE91940.1; AasR: DRN02_008180/QBE91994.1), *Zymomonas mobilis* ZM4 (Aca: ZMO1400/AAV90024.1; Spt: ZMO1270/AAV89894.1; AcpR: ZMO2012/ADK75091.1; Epi: ZMO0205/AAV88829.1; AasR: ZMO0704/AAV89328.2), *Gluconobacter oxydans* 621H (Aca: GOX2417/AAW62148.1; Spt: GOX2056/AAW61792.1; AcpR: GOX2057/AAW61793.1; Epi: GOX1849/AAW61587.1; AasR: GOX2058/AAW61794.1) and *Nitrosomonas eutropha* C91 (Aca: Neut_1100/ABI59355.1; Spt: Neut_0461/ABI58738.1; AcpR: Neut_0460/ABI58737.1; Epi: Neut_0459/ABI58736.1; AasR: Neut_0458/ABI58735.1).

**Table S3.** Possible orthologues for FadD in *G. oxydans*, *N. eutropha* and *C. crescentus*.

| ORF (Query) | ORF (Subject) | E Value | identity (%) | similarity (%) | coverage (%) |
|---|---|---|---|---|---|
| ECD_01775$_{Ec}$ (FadD) | AD932_04675$_{Go}$ | $1e^{-44}$ | 28 | 45 | 85 |
| ECD_01775$_{Ec}$ (FadD) | Neut_1417$_{Ne}$ | $5e^{-60}$ | 29 | 46 | 93 |
| ECD_01775$_{Ec}$ (FadD) | CC_1321$_{Cc}$ | $1e^{-65}$ | 31 | 48 | 91 |
| ECD_01775$_{Ec}$ (FadD) | CC_0966$_{Cc}$ | $3e^{-58}$ | 28 | 45 | 95 |
| SMc02162$_{Sm}$ (FadD) | AD932_04675$_{Go}$ | $3e^{-42}$ | 28 | 44 | 93 |
| SMc02162$_{Sm}$ (FadD) | Neut_1417$_{Ne}$ | $5e^{-38}$ | 27 | 42 | 89 |
| SMc02162$_{Sm}$ (FadD) | CC_0966$_{Cc}$ | $3e^{-60}$ | 29 | 48 | 90 |
| SMc02162$_{Sm}$ (FadD) | CC_1321$_{Cc}$ | $8e^{-56}$ | 31 | 49 | 87 |

Pairwise protein sequence alignments between ORFs codifying for acyl-CoA synthetase (FadD) from *E. coli* ($_{Ec}$) and *S. meliloti* ($_{Sm}$); compared to *G. oxydans* ($_{Go}$), *N. eutropha* ($_{Ne}$) and *C. crescentus* ($_{Cc}$) proteins. Possible homologous ORFs are shown in equal colors. ORF names/accession numbers are as follows: *Caulobacter crescentus* CB15 (CC_1321/AAK23302.1; CC_0966/AAK22950.1), *Escherichia coli* BL21(DE3) (ECD_01775/ACT43629.1), *Gluconobacter oxydans* 621H (AD932_04675/KXV13177), *Nitrosomonas eutropha* C91 (Neut_1417/ABI59664.1) and *Sinorhizobium meliloti* 1021 (SMc02162/CAC41921.1).

**Table S4.** Possible orthologues for AcpR, AcpP, AasR, FadD and Aas proteins in *B. thetaiotaomicron*, *B. stolpii*, *M. xanthus*, *P. gingivalis*, *S. multivorum, S. aurantiaca* and *S. cellulosum*.

| ORF (Query) | ORF (Subject) | E Value | identity (%) | similarity (%) | coverage (%) |
|---|---|---|---|---|---|
| CC_1163$_{Cc}$ (AcpR) | BT_3359$_{Bt}$ | 9e$^{-11}$ | 41 | 51 | 65 |
| CC_1163$_{Cc}$ (AcpR) | C0V70_14770$_{Bs}$ | 7e$^{-07}$ | 29 | 60 | 57 |
| CC_1163$_{Cc}$ (AcpR) | MXAN_6637$_{Mx}$ | 1e$^{-03}$ | 42 | 60 | 51 |
| CC_1163$_{Cc}$ (AcpR) | PGN_1705$_{Pg}$ | 2e$^{-10}$ | 38 | 56 | 60 |
| CC_1163$_{Cc}$ (AcpR) | NCTC11343_01004$_{Sm}$ | 5e$^{-10}$ | 39 | 58 | 55 |
| CC_1163$_{Cc}$ (AcpR) | STAUR_1257$_{Sa}$ | 7e$^{-02}$ | 33 | 60 | 51 |
| CC_1163$_{Cc}$ (AcpR) | sce7052$_{Sc}$ | 1e$^{-09}$ | 44 | 66 | 65 |
| CC_1677$_{Cc}$ (AcpP) | BT_3359$_{Bt}$ | 5e$^{-26}$ | 59 | 74 | 96 |
| CC_1677$_{Cc}$ (AcpP) | C0V70_14770$_{Bs}$ | 3e$^{-14}$ | 42 | 67 | 93 |
| CC_1677$_{Cc}$ (AcpP) | MXAN_4769$_{Mx}$ | 4e$^{-21}$ | 53 | 75 | 93 |
| CC_1677$_{Cc}$ (AcpP) | PGN_1705$_{Pg}$ | 2e$^{-23}$ | 52 | 79 | 93 |
| CC_1677$_{Cc}$ (AcpP) | NCTC11343_01004$_{Sm}$ | 2e$^{-25}$ | 58 | 75 | 93 |
| CC_1677$_{Cc}$ (AcpP) | STAUR_5619$_{Sa}$ | 8e$^{-21}$ | 51 | 75 | 93 |
| CC_1677$_{Cc}$ (AcpP) | sce3814$_{Sc}$ | 2e$^{-20}$ | 56 | 74 | 91 |
| ECD_01090$_{Ec}$ (AcpP) | BT_3359$_{Bt}$ | 6e$^{-27}$ | 62 | 73 | 97 |
| ECD_01090$_{Ec}$ (AcpP) | C0V70_14770$_{Bs}$ | 2e$^{-14}$ | 40 | 65 | 96 |
| ECD_01090$_{Ec}$ (AcpP) | MXAN_4769$_{Mx}$ | 6e$^{-31}$ | 66 | 80 | 97 |
| ECD_01090$_{Ec}$ (AcpP) | PGN_1705$_{Pg}$ | 1e$^{-22}$ | 55 | 75 | 93 |
| ECD_01090$_{Ec}$ (AcpP) | NCTC11343_01004$_{Sm}$ | 3e$^{-24}$ | 62 | 72 | 93 |
| ECD_01090$_{Ec}$ (AcpP) | STAUR_5619$_{Sa}$ | 5e$^{-29}$ | 63 | 78 | 97 |
| ECD_01090$_{Ec}$ (AcpP) | sce3814$_{Sc}$ | 1e$^{-22}$ | 62 | 78 | 84 |
| CC_1165$_{Cc}$ (AasR) | BT_2782$_{Bt}$ | 1e$^{-23}$ | 27 | 45 | 68 |
| CC_1165$_{Cc}$ (AasR) | C0V70_02865$_{Bs}$ | 8e$^{-20}$ | 24 | 40 | 68 |
| CC_1165$_{Cc}$ (AasR) | MXAN_6636$_{Mx}$ | 1e$^{-87}$ | 36 | 52 | 91 |
| CC_1165$_{Cc}$ (AasR) | CF001_1738$_{Pg}$ | 4e$^{-17}$ | 24 | 40 | 79 |
| CC_1165$_{Cc}$ (AasR) | NCTC11343_01971$_{Sm}$ | 1e$^{-13}$ | 24 | 38 | 73 |
| CC_1165$_{Cc}$ (AasR) | STAUR_1258$_{Sa}$ | 3e$^{-92}$ | 36 | 52 | 97 |
| CC_1165$_{Cc}$ (AasR) | sce7053$_{Sc1}$ | 6e$^{-122}$ | 39 | 56 | 96 |
| ECD_01775$_{Ec}$ (FadD) | BT_2782$_{Bt}$ | 4e$^{-63}$ | 30 | 50 | 89 |
| ECD_01775$_{Ec}$ (FadD) | C0V70_02865$_{Bs}$ | 0 | 55 | 74 | 98 |
| ECD_01775$_{Ec}$ (FadD) | MXAN_7148$_{Mx}$ | 6e$^{-79}$ | 33 | 50 | 93 |
| ECD_01775$_{Ec}$ (FadD) | CF001_1738$_{Pg}$ | 2e$^{-29}$ | 25 | 42 | 80 |
| ECD_01775$_{Ec}$ (FadD) | NCTC11343_0197$_{Sm}$ | 8e$^{-37}$ | 28 | 45 | 74 |
| ECD_01775$_{Ec}$ (FadD) | STAUR_3279$_{Sa}$ | 0 | 58 | 73 | 98 |
| ECD_01775$_{Ec}$ (FadD) | sce3825$_{Sc}$ | 5e$^{-54}$ | 32 | 47 | 94 |
| LA59_RS23465$_{Vh}$ (Aas) | BT_2782$_{Bt}$ | 7e$^{-38}$ | 25 | 42 | 95 |
| LA59_RS23465$_{Vh}$ (Aas) | C0V70_10230$_{Bs}$ | 2e$^{-44}$ | 27 | 45 | 93 |
| LA59_RS23465$_{Vh}$ (Aas) | MXAN_6374$_{Mx}$ | 4e$^{-115}$ | 36 | 55 | 97 |
| LA59_RS23465$_{Vh}$ (Aas) | CF001_1738$_{Pg}$ | 1e$^{-15}$ | 22 | 40 | 75 |
| LA59_RS23465$_{Vh}$ (Aas) | NCTC11034_01400$_{Sm}$ | 3e$^{-28}$ | 23 | 42 | 79 |
| LA59_RS23465$_{Vh}$ (Aas) | STAUR_2914$_{Sa}$ | 2e$^{-112}$ | 37 | 55 | 97 |
| LA59_RS23465$_{Vh}$ (Aas) | sce5736$_{Sc}$ | 1e$^{-107}$ | 36 | 52 | 97 |

Pairwise protein sequence alignments between ORFs codifying for: acyl carrier protein (AcpR) and acyl-ACP synthetase (AasR) from *C. crescentus* CB15 ($_{Cc}$), acyl-CoA synthetase (FadD) from *E. coli* BL21(DE3) ($_{Ec}$), and acyl-ACP synthetase (Aas) from *V. harveyi* ($_{Vh}$); compared to the *B. thetaiotaomicron* ($_{Bt}$), *B. stolpii* ($_{Bs}$), *M. xanthus* ($_{Mx}$), *P. gingivalis* ($_{Pg}$), *S. multivorum* ($_{Sm}$), *S.*

*aurantiaca* ($_{Sa}$) and *S. cellulosum* ($_{Sc}$) proteins. Possible homologous ORFs are shown in equal colors. ORF names/accession numbers are as follows: *Bacteroides thetaiotaomicron* VPI-5482 (BT_3359/Q8A2E6.1; BT_2782/AAO77888), *Bacteriovorax stolpii* (C0V70_14770/AUN99345.1; C0V70_02865/AUN97064; C0V70_10230/AUN98475), *Myxococcus xanthus* DK 1622 (MXAN_6637/ABF91660.1; MXAN_4769/ABF90332.1; MXAN_6636/ABF92798; MXAN_7148/ABF89596; MXAN_6374/ABF88458), *Porphyromonas gingivalis* ATCC 33277 (PGN_1705/B2RLH9.1; CF001_1738/AUR49213), *Sphingobacterium multivorum* (NCTC11343_01004/SPZ84464.1; NCTC11343_01971/SPZ85409; NCTC11034_01400/SUJ04263), *Stigmatella aurantiaca* DW4/3-1 (STAUR_1257/ADO69061.1; STAUR_5619/ADO73384.1; STAUR_1258/ADO69062; STAUR_3279/ADO71071; STAUR_2914/ADO70706) and *Sorangium cellulosum* So ce56 (sce7052/CAN97221.1; sce3814/CAN93974.1; sce7053/CAN97222.1; sce3825/CAN93985.1; sce5736/CAN95899.1)

**Table S5**. Oligonucleotides used for amplification of different sphingolipid biosynthesis genes. Sites for recognition by restriction enzymes are underlined.

| Primers | Sequence (5´-3´) |
|---|---|
| **Primers for expression plasmids** | |
| oLOP227 | AGGAATACATATGATCACGGCCACGGCGTC |
| oLOP228 | AAAGGTACCTCAGTCGCCATAGGTTCCGGC |
| oLOP264 | AGGAATACATATGTCCGACATTCTCGAGCG |
| oLOP265 | AAAGGATCCTTAGGCGGTCTTTTCCGTG |
| oLOP423 | ACTGGGTACCCATATGGGGCTATTTGATAAGCACC |
| oLOP424 | ACTGTCTAGAGGATCCTCAGGCGCGGGC |
| oLOP432 | ACTGCATATGGTAAATCGTGAAATAGTAATG |
| oLOP433 | ACTGGGATCCTTATTTATTCTCCAGCCATG |
| oLOP434 | ACTGACTGCCATGGCATATGGGGCTATACGATAAATATGCGC |
| oLOP435 | ACTGTCTAGAGGATCCTTACTGCTTCAGAGTAGCAAATGCCTGA |
| oLOP436 | ACTGACTGCCATGGCATATGGCCGACCTCCTCTCCAAG |
| oLOP437 | ACTGTCTAGAGGATCCTCAGGGGATTACTCCCGTGGCG |
| oLOP438 | ACTGGATATCATGGGGCTATTTGATAAGCACCTGG |
| oLOP439 | ACTGGGCCGGCCTCAGGCGCGGGCGCG |
| oLOP440 | ACTGCATATGGTGTATATGTCTAATAAAATC |
| oLOP443 | ACTGCTCGAGTCATGCCAGGGATTCC |

**Table S6. Construction of different expression plasmids**
For details see Materials and methods in main text.

| Plasmid | ORFs cloned | oligonucleotides used | restricted with | cloned into plasmid restricted with () |
|---|---|---|---|---|
| pDG04 | CC_1165 | oLOP227/oLOP228 | *Nde*I/*Kpn*I | pET17b (*Nde*I/*Kpn*I) |
| pPEG01 | CC_1677 | oLOP264/oLOP265 | *Nde*I/*Bam*HI | pET9a (*Nde*I/*Bam*HI) |
| pJPG05 | CC_1162 | oLOP423/oLOP424 | *Kpn*I/*Xba*I | pBAD24 (*Kpn*I/*Xba*I) |
| pJPG06 | ECD_02854 | oLOP434/oLOP435 | *Nco*I/*Xba*I | pBAD24 (*Nco*I/*Xba*I) |
| pJPG07 | Swit_3900 | oLOP436/oLOP437 | *Nco*I/*Xba*I | pBAD24 (*Nco*I/*Xba*I) |
| pJPG12 | ECD_02853 | oLOP432/oLOP433 | *Nde*I/*Bam*HI | pET9a (*Nde*I/*Bam*HI) |
| pJPG15 | CC_1162 | oLOP438/oLOP439 | *Eco*RV/*Fse*I | pJPG13 (MCS-2) (*Eco*RV/*Fse*I) |
| pJPG17 | ECD_02851 | oLOP440/oLOP443 | *Nde*I/*Xho*I | pET17b (*Nde*I/*Xho*I) |
| pJPG20 | ECD_02853/ ECD_02854 | oLOP432/oLOP435 | *Nde*I/*Bam*HI | pCDFDuet-1 (MCS-2) (*Nde*I/*Bgl*II) |
